# Transcriptional approach to study porcine tracheal epithelial cells individually or dually infected with swine influenza virus and *Streptococcus suis*

**DOI:** 10.1186/1746-6148-10-86

**Published:** 2014-04-07

**Authors:** Yuan Dang, Claude Lachance, Yingchao Wang, Carl A Gagnon, Christian Savard, Mariela Segura, Daniel Grenier, Marcelo Gottschalk

**Affiliations:** 1 Faculté de Médecine Vétérinaire, Université de Montréal, 3200 Sicotte, St-Hyacinthe, J2S 2M2 Québec, Canada; 2 Groupe de Recherche en Écologie Buccale, Faculté de Médecine Dentaire, Université Laval, G1V 0A6 Québec City, Canada

**Keywords:** *Streptococcus suis*, Swine Influenza virus, Co-infection, Microarray, Cytokines/chemokines induction, Porcine tracheal epithelial cells

## Abstract

**Background:**

Swine influenza is a highly contagious viral infection in pigs affecting the respiratory tract that can have significant economic impacts. *Streptococcus suis* serotype 2 is one of the most important post-weaning bacterial pathogens in swine causing different infections, including pneumonia. Both pathogens are important contributors to the porcine respiratory disease complex. Outbreaks of swine influenza virus with a significant level of co-infections due to *S. suis* have lately been reported. In order to analyze, for the first time, the transcriptional host response of swine tracheal epithelial (NPTr) cells to H1N1 swine influenza virus (swH1N1) infection, *S. suis* serotype 2 infection and a dual infection, we carried out a comprehensive gene expression profiling using a microarray approach.

**Results:**

Gene clustering showed that the swH1N1 and swH1N1/*S. suis* infections modified the expression of genes in a similar manner. Additionally, infection of NPTr cells by *S. suis* alone resulted in fewer differentially expressed genes compared to mock-infected cells. However, some important genes coding for inflammatory mediators such as chemokines, interleukins, cell adhesion molecules, and eicosanoids were significantly upregulated in the presence of both pathogens compared to infection with each pathogen individually. This synergy may be the consequence, at least in part, of an increased bacterial adhesion/invasion of epithelial cells previously infected by swH1N1, as recently reported.

**Conclusion:**

Influenza virus would replicate in the respiratory epithelium and induce an inflammatory infiltrate comprised of mononuclear cells and neutrophils. In a co-infection situation, although these cells would be unable to phagocyte and kill *S. suis*, they are highly activated by this pathogen. *S. suis* is not considered a primary pulmonary pathogen, but an exacerbated production of proinflammatory mediators during a co-infection with influenza virus may be important in the pathogenesis and clinical outcome of *S. suis*-induced respiratory diseases.

## Background

*Streptococcus suis* is one of the most important post-weaning bacterial pathogens in swine causing mainly septicemia with or without sudden death, meningitis, arthritis and endocarditis. It is also considered an agent of pneumonia, although its role as primary or secondary respiratory pathogen has been controversial [1]. Over the last few years, this pathogen has been considered an emerging zoonotic agent [2]. Human infections with *S. suis* manifest mainly as meningitis, septicemia and septic shock [3]. Among the described *S. suis* serotypes, type 2 is usually considered as the most virulent for both pigs and humans in most countries [2]. Pigs usually acquire *S. suis* via the respiratory route [1]. In fact, colonization of the nasopharyngeal cavity is an important risk factor for *S. suis* infection of piglets. Some colonized animals may never develop disease (carrier animals); on the other hand, some carrier piglets will eventually develop bacteremia, with dissemination in the bloodstream followed by septicemia [1]. It is believed that humans can become infected through skin lesions, surface mucosa and/or the oral route following the ingestion of contaminated pork products [4]. Tonsil carriage of *S. suis* by humans without clinical signs (usually slaughterhouse workers) has also been described [5,6].

Although there is evidence suggesting that the nasopharynx and palatine tonsils may be the routes of entry in swine invasive diseases [7], it is still unknown how virulent serotype 2 strains of *S. suis* manage to cross the first natural line of the host defense to initiate disease. It has been suggested that the pathogen would breach the mucosal epithelium in the upper respiratory tract, locally contributing to respiratory pathology and/or further invading the bloodstream [8]. Limited data are available concerning the interaction between *S. suis* and swine respiratory epithelial cells. Ferrando and colleagues [9] described *S. suis* adhesion (but not invasion) to porcine tracheal epithelial cells. More specifically, bacterial adherence was 20-fold stronger than that previously reported with the human laryngeal carcinoma cell line HEp-2.

Swine influenza is a highly contagious viral infection in pigs affecting the respiratory tract that can have significant economic impacts [10]. Although this infection is typically self-limited with high-morbidity but low mortality, secondary complications substantially increase illness and death [11]. In fact, swine influenza virus is a key contributor to the porcine respiratory disease complex (PRDC), a multifactorial syndrome characterized by severe respiratory disease after infection with two or more infectious agents. Both *S. suis* and swine influenza virus are part of the PRDC. Outbreaks of swine influenza virus with a significant level of co-infections with *S. suis* have been lately reported in England [12]. More recently, we have shown an increased adhesion/invasion of *S. suis* serotype 2 in influenza pre-infected tracheal epithelial cells [13]. Preliminary studies suggested increased activation of co-infected tracheal epithelial cells [13].

No data were available so far concerning a complete transcriptional response of swine epithelial cells to swine influenza virus infection, *S. suis* infection and a dual infection. Therefore, we carried out a comprehensive gene expression profiling of H1N1 virus infection, *S. suis* serotype 2 bacterial infection and dual virus-bacterial infection of swine tracheal epithelial cells using a microarray approach. Results showed that cells are highly activated after 24 h incubation with influenza virus and, to a lesser extent, after 12 h incubation with *S. suis*. However, bacterial infection of previously virus-infected cells showed a clear synergy with an increased expression of certain inflammatory-related genes. An increased inflammation in the lungs in the presence of both pathogens may lead to a more serious respiratory disease syndrome in pigs and may explain, at least in part, the contribution of *S. suis* to pneumonia as a secondary pathogen.

## Results

### Transcriptional response of tracheal epithelial cells infected by swine influenza virus, *S. suis* or both pathogens

To analyze the early transcriptional response following infection with *S. suis* of swine influenza virus-infected or non-infected porcine tracheal epithelial cells, an Agilent porcine microarray assay was carried out. Cells were also analyzed in the presence of the virus only. In co-infected experiments, cells were pre-infected with a H1N1 strain of swine influenza virus for 12 h and then further incubated for 12 h with a virulent strain of *S. suis* serotype 2. Virus replication in these epithelial cells was observed during the first 12 h of incubation, reaching a plateau that lasted for the following 12 h [13]. Cells infected with virus alone, bacteria alone, or co-infected with both pathogens did not present any significant levels of cytotoxicity (lower than 5%, data not shown).

**Figure 1:**
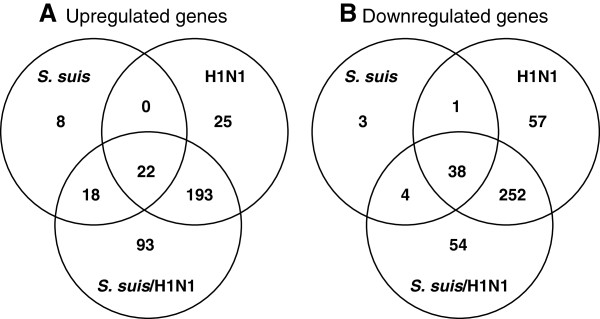
**Host genes are modified in greater numbers in NPTr cells co-infected by *S. suis *and swine influenza virus.** Venn diagram showing the total numbers of upregulated **(A)** or downregulated **(B)** genes in NPTr cells (*n* = 4 replicates per group) infected with *S. suis*, H1N1, or co-infected with *S. suis*/H1N1 when compared to mock-infected cells, as determined by Agilent microarray study. Differentially expressed genes were defined by fold changes greater than 2-folds (upregulation or downregulation) with an accompanying *p*-value ≤ 0.05.

**Figure 2:**
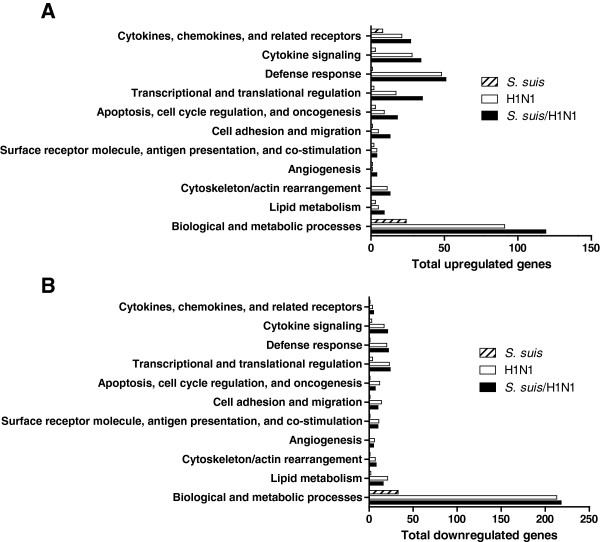
**Distribution and functional categories of genes differentially expressed by infected NPTr cells.** Distribution of upregulated **(A)** or downregulated **(B)** genes into different biologic functional categories from NPTr cells infected with *S. suis*, H1N1, or co-infected with *S. suis*/H1N1 when compared to mock-infected cells, as determined by Agilent microarray study.

Data from activated cells were compared to mock-infected cells. Using an expression threshold ≥ 2 fold with a *p* < 0.05, a total of 588, 96 and 673 transcripts were modified by the swH1N1 alone, *S. suis* alone, or both pathogens in co-infection, respectively. Of them, some upregulated and downregulated genes were shared by the two pathogens (Figures 1A and B). The complete list of genes is shown in Additional file 1: Tables S1 and S2. These genes were widespread within different biologic functional categories (Figure 2). Particularly, and other than genes related to biological and metabolism processes, genes associated with immune and inflammatory response were highly overexpressed among upregulated genes, which indicate that they may play important roles not only in host defense but also in pathology (inflammation). It was clear that a pre-infection with swH1N1 for 12 h caused a higher impact on *S. suis* modulation of mRNA expression compared to cells not previously infected with the virus (Additional file 1: Tables S1 and S2).

**Figure 3:**
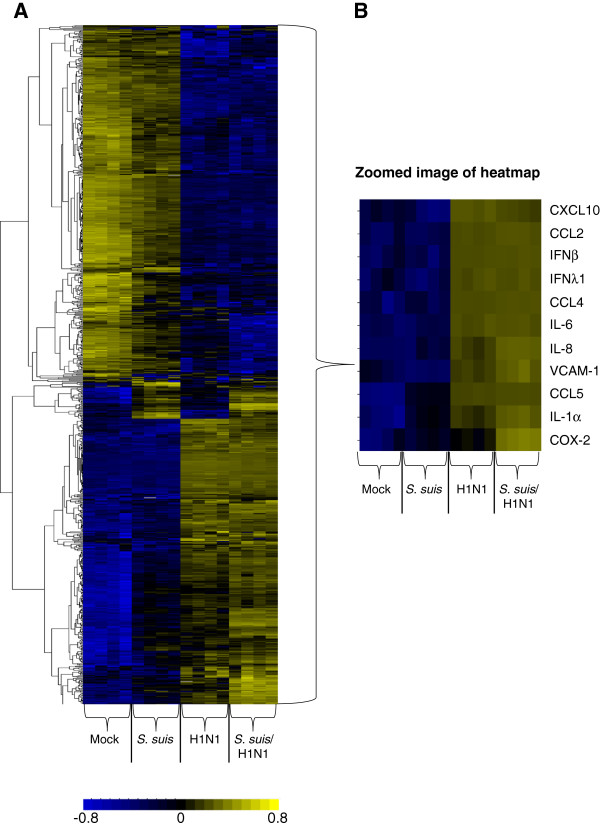
**Heat map of hierarchical clustering analysis of differentially expressed genes in infected NPTr cells.** Genes differentially expressed were subjected to hierarchical clustering (complete linkage method) using uncentered correlation as similarity measure. **(A)** Horizontal rows represent individual genes and vertical columns represent individual samples from mock-infected, *S. suis*, H1N1, *S. suis*/H1N1 (*n* = 4). **(B)** Interesting genes are zoomed in from the heat map in **A**. Color scale: black, average signal intensity; brightest blue and brightest yellow indicate low and high expression levels, respectively.

Data from the microarray were further analyzed in order to compare clustering of genes in NPTr cells differently infected with either pathogen alone or together. An unsupervised hierarchical clustering of differentially expressed genes was performed (Figure 3A). Clustering of the genes showed that infection with swH1N1 alone and with both pathogens (swH1N1/*S. suis*) highly modified the expression of genes. Additionally, infection of NPTr cells by *S. suis* resulted, after 12 h of incubation, in a lower but still significant levels of expressed genes compared to mock-infected cells. A few groups of genes were upregulated similarly in cells infected by *S. suis* alone or in co-infected cells without any impact resulting from virus infection.

Interesting genes that were significantly upregulated are zoomed in (Figure 3B) to better analyze their expression pattern in differently infected NPTr epithelial cells. For example, results showed that swH1N1 infection significantly increased the expression of the chemokines CCL2 (MCP-1), CCL4 (MIP-1β), CCL5 (RANTES), IL-8 and CXCL10 (IP-10) (Additional file 1: Table S1). In the case of the monocyte chemoattractants CCL2, CCL4 and CCL5, their mRNA expression increase was particularly important. Of these three genes, only CCL5 was significantly up-regulated (to a much lesser extent when compared to swH1N1 infection) by *S. suis* alone. Similarly, the proinflammatory cytokines IL-1α and IL-6 were mainly up-regulated by the swH1N1 infection. In addition, genes related to cell adhesion and migration, such as the β_1_ integrin vascular cell adhesion molecule 1 (VCAM-1), were also up-regulated by virus infection. An important lipid metabolic mediator, cyclooxygenase (COX)-2 mRNA, was up-regulated by virus alone and both pathogens together. Plasminogen activator urokinase (PLAU), a protease involved in degradation of the extracellular matrix, was upregulated by virus infection and coinfection with *S. suis* increased this upregulation (Additional file 1: Table S1).

As expected, swH1N1 infection alone only clearly up-regulated genes coding for interferon and interferon-regulatory factors. More precisely, type I interferons (IFN) and, to a certain extent, type III IFN (IFNλ1) gene expression were increased (Additional file 1: Table S1). Of the type I IFNs, IFNβ gene but not IFNα gene was up-regulated. IFN-regulated/stimulated genes affected by virus infection are presented in detail in Additional file 1: Table S1.

### Quantitative RT-PCR results

**Figure 4:**
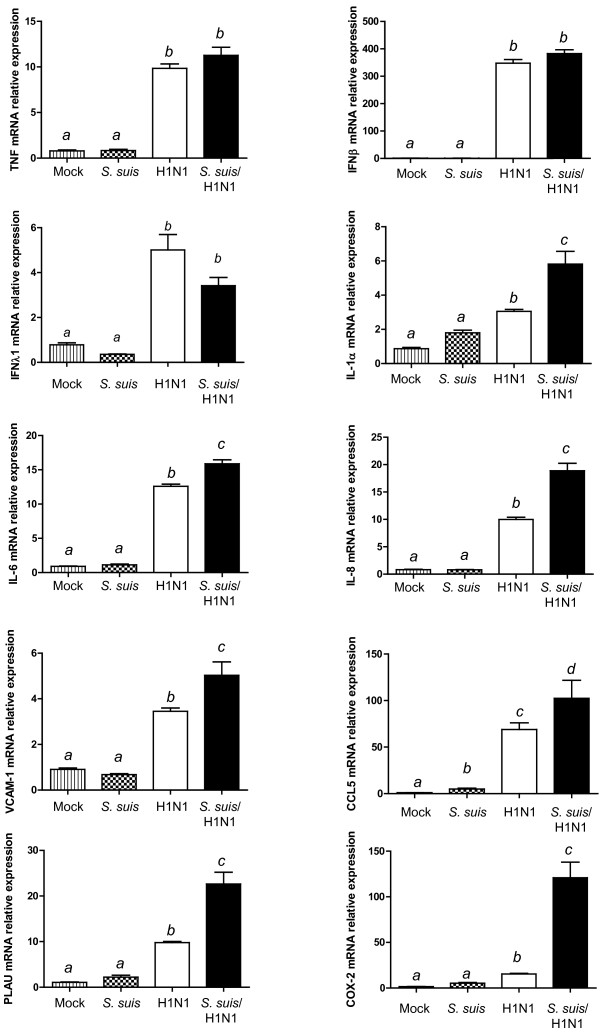
**Proinflammatory cytokine and chemokine genes are highly expressed in NPTr epithelial cells infected by *S. suis *and swine influenza virus.** Expression of TNF, IFN-β, IFNλ1, IL-1, IL-6, IL-8, VCAM-1, CCL5, PLAU, and COX-2 genes in NPTr cells infected with *S. suis*, H1N1, or *S. suis*/H1N1 when compared to mock-infected cells, as quantified by qPCR assay. Data represent mean relative expression values of mRNA ± SEM. Groups that are significantly different are indicated by different letters (*a*, *b*, and *c*), as determined by One-way ANOVA with *p* ≤ 0.05.

In order not only to validate microarray results but also to further study some specific genes based on their potential implication in immune and inflammatory response processes, we carried out quantitative real-time PCR (qPCR) on 13 different genes (Additional file 1: Table S3). All tested genes presented a perfect correlation with microarray results, with the exception of TNF. This proinflammatory cytokine did not show any significant difference by microarray, but its expression was shown to be up-regulated for virus and co-infected cells (Figure 4). A higher sensitivity of the qPCR assay can explain this difference.

Results obtained with qPCR assay for IFN gene expression confirmed those obtained by microarray. As expected, virus infection was responsible for type I and III IFN up-regulation. IFNβ was up-regulated to a much greater extent than IFNλ1. On the other hand, qPCR results of genes coding for IL-1α, IL-6, IL-8 and VCAM-1 confirmed no activation by *S. suis* alone, an intermediate upregulation by swH1N1 infection but a significant increase when cells were co-infected with both pathogens (Figure 4 and Additional file 1: Table S3). Interestingly, a clear increased of IL-6 and IL-8 by *S. suis* was reported in the literature using the same cells [13]. However, in that study, a late bacterial incubation time was used (24 h), whereas an early time response (12 h) was analyzed in the present study. The chemokine CCL5 was upregulated by *S. suis*, but considerably more activated by virus and co-infection, as suggested by microarray results. Microarray results obtained with PLAU and COX2 were also confirmed by qPCR with a very significant upregulation of both gene expressions in co-infected cells (Figure 4 and Additional file 1: Table S1).

## Discussion

Airway epithelial cells are the initial site of both influenza virus and *S. suis* infections [1,14]. Interestingly, *S. suis* is not considered a primary cause of swine pneumonia, but rather a secondary respiratory pathogen that would complicate viral infections [1]. In a recent study, tracheal cells pre-infected with swH1N1 showed a significant increase of *S. suis* adhesion and invasion levels when compared to normal cells. In fact, it was shown that the bacterial capsular sialic acid moiety was responsible for the increased binding of *S. suis* to the influenza virus hemagglutinin protein expressed on the surface of virus-infected cells [13]. A comprehensive host gene expression profile of these cells infected with either swine influenza virus, *S. suis*, or both pathogens (co-infection) has not been reported so far.

The innate immune response is the first line of defense against influenza infection. Among innate responses, types I (IFNα and IFNβ) and III (IFNλ) IFN induction and signaling are potent protection mechanisms against viral infections [15]. Type III IFNs and IFNβ, which contains similar promoter elements, are known to be the first IFNs upregulated in response to pattern recognition receptor signaling, while IFNα gene expression is driven as part of IFN signaling amplification [16]. Both IFNβ and, to a lesser extent, IFNλ1 were significantly induced by swH1N1 virus only. As previously described [15], no clear upregulation of IFNα was observed. *In vivo*, IFNα is known to be an abundant and important cytokine during influenza infection, but most often this finding is linked to its expression in serum, probably produced by plasmacytoid dendritic cells [17]. Interestingly, some genes were downregulated in the presence of virus infection. Since it has been shown that non-structural (NS) proteins of influenza may modulate and down-regulate host-cellular processes, a certain role of such NS proteins cannot be completely excluded [18].

Influenza virus infection at the respiratory tract site is also characterized by an early influx of neutrophils followed by an increased recruitment of blood derived monocytes within the first days of infection. This influx is driven by the release of chemokines from infected epithelial cells and alveolar macrophages [19]. In the present study, virus infection significantly increased the expression of important chemokines in epithelial cells. Of these genes, only CCL5 was induced by bacteria after 12 h of incubation in the absence of virus infection. However, as it was the case for IL-6 and IL-8, CCL5 expression was clearly higher in co-infected cells, probably caused by an additive and/or synergistic effect due to the simultaneous presence of both pathogens. Interestingly, it has already been reported that *S. suis* is able to induce the production of CCL5 *in vivo* and *in vitro* from phagocytic and choroid epithelial cells [20,21]. In addition, since an increased *S. suis* adhesion/invasion of tracheal cells is observed in the presence of influenza virus [13], the increased expression of some cytokines may be the consequence of higher bacteria to cell ratio.

It has been reported that epithelial cells infected by influenza virus elicit trans-epithelial recruitment of monocytes mainly in a CCL2-dependent manner that is primarily reliant on the engagement of VCAM-1 [19]. In our study, expression of both CCL2 and VCAM-1 genes was significantly increased after co-infection of swine epithelial cells. Although we could not detect a clear increase of VCAM-1 expression by *S. suis* infection alone, a potential role of this pathogen in co-infected cells cannot be ruled out. Expression of VCAM-1 gene was shown in porcine choroid plexus epithelial cells activated by *S. suis*[22] and in different tissues after *in vivo* infection [23]. In addition, it has been shown that *S. suis* induces the upregulation of intercellular adhesion molecule-1, CD11a/CD18, and CD11c/CD18 expression on human monocytes [24]. As mentioned, a higher bacterial charge (in the presence of influenza virus infection) [13] could also further activate epithelial cells. The accumulation of large numbers of monocytes within the lung parenchyma and alveolar spaces has been described as a hallmark of early stages of influenza virus infection [10], and it may be amplified when *S. suis* is also present. Recently, studies using cultured human lung organ and bronchial/tracheal epithelial cells showed that an influenza virus infection induces considerable amount of CXCL10, a chemokine that attracts activated natural killer and Th1 cells, which have an essential role in virus infection clearance [25]. Although we have previously reported that *S. suis* induces the production of CXCL10 from dendritic cells [26], gene upregulation in tracheal cells were only observed with swH1N1 infection in the present study.

Eicosanoids are lipid mediators derived from arachidonic acid that play critical roles in the host response to infection [27]. The cyclooxygenase enzymes (COX-1 and COX-2), which catalyze the first step in the biosynthesis of prostaglandins (PGs) from arachidonic acid, have specifically been implicated in host response to infection [27]. One of the most important PGs is PGE_2_, which is known to contribute to excessive inflammation. COX-1 is generally believed to be constitutively expressed whereas COX-2 is the inducible form [28]. COX-2 induction is caused by a variety of stimuli, including bacteria and virus, and it has been shown to play an important role in pneumonia as previously demonstrated for influenza and *Streptococcus pneumoniae* infections [29-31]. In swine veterinary infections, COX-2 plays an important role in lung inflammation in animals experimentally infected with *Actinobacillus pleuropneumoniae*[29]. Very little is known about the activation of lipid metabolism by *S. suis*. There was only one report showing that *S. suis* is able to induce the secretion of PGE_2_ by human macrophages [32]. More recently, we have shown that *S. suis* infection is accompanied by an increase of arachidonic acid, a proinflammatory ω-6 polyunsaturated fatty acids (PUFA), and by a decrease of docosahexaenoic acid, an anti-inflammatory ω-3 PUFA. Macrophages infected with *S. suis* showed activation of mitogen-activated protein kinase pathways and COX-2 increased expression [33]. In the present study, the expression of COX-2 by tracheal epithelial cells infected with both pathogens was 25 folds higher than that observed with virus alone, indicating a synergistic effect of *S. suis* on lipid metabolism. These data seem to indicate that lipid mediators play an important role in *S. suis* systemic disease as well as in influenza/*S. suis* co-infections at the respiratory tract level.

Finally, virus induced the expression of PLAU. This induction was significantly upregulated in the presence of both pathogens and it can have important pathological consequences. In fact, we have previously shown that *S. suis* specifically binds porcine plasminogen on its surface [34]. Once bound, plasminogen can be converted into proteolytically active plasmin by urokinase plasminogen activator [34]. Plasmin-coated *S. suis* has been reported to induce fibronectin degradation [34], a phenomenon that may contribute to pathogen dissemination into tissues as well as to the inflammatory process, given that certain fibronectin fragments are known to be chemotactic for monocytes [35] and can induce cytokine secretion by macrophages [36].

Finally, it is important to note that results obtained in the present study were generated with a typical European ST1 strain of *S. suis*. Serotype 2 strains present different genotypic and phenotypic characteristics depending on the geographical area [2]. Although strains from different origins and different virulence properties have been shown to similarly adhere and invade epithelial cells infected with H1N1 virus [13], host cell activation using strains from different geographical origins should be confirmed.

## Conclusion

We carried out, for the first time, a comprehensive gene expression profiling study using a microarray approach on swine tracheal epithelial cells infected by either swine influenza virus, *S. suis*, or both (co-infection). Pro-inflammatory genes (cytokines and chemokines) but also those related to lipid metabolism playing a role in inflammation were significantly upregulated during co-infection. Upregulation of certain genes involved in bacterial pathogenesis could also influence a higher virulence of *S. suis* infection in the presence of influenza virus. It is important to mention that these results were obtained from *in vitro* assays and they should be confirmed with *in vivo* studies. The observed synergistic effect may also be the consequence, at least in part, of a higher *S. suis* adhesion to the virus-hemagglutinin expressed by epithelial cells previously infected with swine influenza virus. In a co-infection situation, influenza virus would replicate in the respiratory epithelium inducing an inflammatory infiltrate comprised of neutrophils and mononuclear cells. Despite that these cells are unable to phagocyte and kill *S. suis*, they are highly activated by this pathogen. *S. suis* is not considered a primary pulmonary pathogen, but an exacerbated local production of proinflammatory mediators during a co-infection with influenza virus, as strongly suggested by the results obtained in the present study, may be important in the pathogenesis and clinical outcome of *S. suis* associated respiratory infections.

## Methods

### Bacterial strains, epithelial cells and influenza virus strain

The well characterized *S. suis* strain 31533, which is a highly virulent European strain isolated from a diseased pig was used throughout this study [37,38]. This strain is a serotype 2, sequence type 1 (as determined by multilocus sequence typing), and well encapsulated under culture and assay conditions used in this study. Bacterial growth conditions were performed as previously reported, with some modifications [38]. Briefly, *S. suis* was grown overnight on Todd-Hewitt agar (THA) (Becton Dickinson, Mississauga, ON, Canada). Isolated colonies were used as inoculum in 5 ml of Todd-Hewitt broth (THB; Becton Dickinson) and incubated during 8 h at 37°C with agitation. Working bacterial cultures for epithelial cells infections were produced by inoculating 10 μl of a 1000 fold serial dilution into 30 ml THB with agitation at 37°C for 16 h. Bacteria were washed three times in phosphate-buffered saline (PBS, pH 7.3) and appropriately diluted in cell culture medium for the experiments. The number of CFU/ml in the final suspension was determined by plating samples onto THA using an Autoplate® 4000 Automated Spiral Plater (Spiral Biotech, Norwood, MA).

The pig tracheal epithelial cell line (NPTr) [39] was used for virus growth and co-infection studies. NPTr cells were grown in Minimum Essential Medium (MEM; Invitrogen, Burlington, ON, Canada) supplemented with 10% (v/v) heat-inactivated fetal bovine serum (FBS), penicillin-streptomycin (100 U/ml), gentamycin (0.04 mg/ml), sodium pyruvate (1 mM), and L-glutamine (2 mM; Invitrogen). Cells were cultured at 37°C with 5% CO_2_ in a humid atmosphere in T75 flasks or 24-well tissue culture plates (Falcon; Becton Dickinson). For assays, cells were treated with 0.05% trypsin in 0.03% EDTA solution and diluted in culture medium to obtain a final concentration of 10^5^ cells/ml. Then, the cell suspension was distributed into tissue culture plates and incubated until cell confluence was reached. Twenty-four hours before the assays, culture medium was removed from the wells and replaced with fresh complete medium without antibiotics.

Swine influenza virus H1N1 (swH1N1, strain A/swine/St-Hyacinthe/148/1990) isolated from a case of swine flu in Canada was also used for this study [40]. Virus was propagated in NPTr cells as described [39]. Aliquots of the supernatant containing infectious virions were stored at −70°C. The titer of the produced viral stock was 10^7.25^ TCID_50_/ml.

### NPTr co-infection by swine influenza virus and *S. suis*

The methodology used was similar to the one recently described [13]. There were 4 groups: a) non-infected cells (24 h, control); b) cells infected with virus alone (24 h); c) mock-infected cells (12 h) and then infected with *S. suis* (12 h); and d) virus-infected cells (12 h) and then infected with *S. suis* (co-infection). Four replicates of independent experiments were done for microarray analysis. The incubation time (12 h) for *S. suis* was chosen to evaluate the early response, mainly in co-infected cells. For the virus infected cells, swH1N1 (MOI:1) was inoculated onto NPTr cell monolayers in 24-well culture plates and incubated for 1 h with antibiotic free MEM at 37°C in 5% CO_2_. The virus-infected cells were then washed twice with PBS and fresh media containing 10% FBS without antibiotic was added and incubated for 12 h (for the co-infected group) or 24 h for virus infected group. Infectious viral load profile was determined by virus titration in cell cultures and RT-PCR.

For the co-infected group, after a 12 h incubation at 37°C in 5% CO_2_ with virus, cells were further infected with *S. suis* (10^6^ CFU/well, MOI:10). Plates were centrifuged at 800 × *g* for 10 min in order to bring bacteria in close contact with the cells [41], and incubated at 37°C in 5% CO_2_ for 12 h. Similar bacterial treatment was done to cells which were not previously infected by virus (control). Low cell toxicity levels were confirmed using Cytotox 96 kit (Promega, Madison, WI) from culture supernatants according to manufacturer’s instructions. In parallel experiments, cells were either mock-infected, infected with either virus or *S. suis* alone following a similar methodology as described above.

### Cell collection, homogenization and extraction of total RNA

At 12 h post-infection with *S. suis,* media was removed and cells were washed once with PBS. Cells were then treated with a lysis solution (RLT solution; Qiagen, Valencia, CA) and total RNA from homogenized cells was isolated and purified using RNeasy mini kit with on-column DNase digestion according to manufacturer’s protocol (Qiagen). Total RNA was kept at −80°C.

### Agilent microarray analysis

The microarray experiment was performed at the McGill University and Genome Québec Innovation Centre (Montréal, Québec, Canada) using the Agilent porcine (v2) gene expression microarray 4x44K (Agilent Technologies, Santa Clara, CA). Prior to the microarray experiment, total RNA quality and quantity was assessed using the Agilent 2100 Bioanalyzer (Agilent Technologies). The microarray was performed according to the manufacturer’s instructions. Samples positions on chip were randomly distributed.

### Microarray data accession number

All microarray raw data are available and have been deposited in the Gene Omnibus Expression database under accession numbers GSE52172.

### Validation of microarray data by RT-qPCR

Thirteen genes were selected for the validation of microarray results by RT-qPCR (Table 1). The qPCR analysis was executed to conform to the qPCR MIQE guidelines [42]. The validation experiments were performed using the same RNA samples that were used for the microarray study and other supplemental samples. Extracted RNA was converted into cDNA by reverse transcription of 500 ng total RNA using a Quantitect cDNA synthesis kit (Qiagen). Then, qPCR assays were carried out using SsoFast Evagreen Supermix kit (Bio-Rad, Hercules, CA) and gene-specific primers (250 nM) on a CFX96 rapid thermal cycler system (Bio-Rad). The cycling conditions were: 3 min of polymerase activation at 98°C followed by 40 cycles at 98°C for 2 s and 57°C for 5 s. Melting curves were generated after each run to confirm a single PCR product.

**Table 1:** Primer sequences used for real-time RT-qPCR

**Gene**	**Genebank ID**	**Amplicon size**	**Forward sequence (primer #)**	**Reverse sequence (primer #)**	**Efficiency (qPCR)**
***Hipox***	NM_001032376	142 bp	GCAGCCCCAGCGTCGTGATT	CGAGCAAGCCGTTCAGTCCTGT	99
***Ppia***	NM_214353	133 bp	TGCAGACAAAGTTCCAAAGACAG	GCCACCAGTGCCATTATGG	97
***Ccl5***	NM_001129946	78 bp	GAAATGGGTGCGGGAGTACA	GTTTGCACGAGTTCAGGCTC	94
***Cox2***	NM_214321	165 bp	TAGGATTCAGGGCTTTCACTGGCT	TGTCAGCCGACAATGAGATGTGGA	105
***Ifn***β	NM_001003923	150 bp	TGCAACCACCACAATTCCAGAAGG	TCTGCCCATCAAGTTCCACAAGGA	102
***Il1a***	NM_214029	152 bp	TGAAGATGGCCAAAGTCCCTGACCT	ATCCATGCCGTCCCCAGGAAGTG	93
***Il6***	NM_214399	105 bp	ACTCCCTCTCCACAAGCGCCTT	TGGCATCTTCTTCCAGGCGTCCC	97
***Il8***	NM_213867	80 bp	TGTGAGGCTGCAGTTCTGGCAAG	GGGTGGAAAGGTGTGGAATGCGT	95
***Il12a***	NM_213993	162 bp	CTGAAGGCCGTCAGCAACAC	AGCCAGGCAACTCTCATTCG	92
***Ifn***λ***1***	NM_001142837	135 bp	TGGCCTTAGAGGCTGAGCTA	CCCTGATGCAAGCCTGAAGT	91
***Irf1***	NM_001097413	113 bp	AATCCAGCCCTGATACCTTCTCT	GGCCTGTTCAATGTCCAAGTC	95
***Irf7***	NM_001097428	94 bp	CTGCGATGGCTGGATGAA	TAAAGATGCGCGAGTCGGA	90
***Plau***	NM_213945	143 bp	CTCCAAAGGCAGCCATGAAC	CACAGTGCTCCCCTTGGAAT	106
***Tnf***	NM_214022	112 bp	GCCACCACGCTCTTCTGCCTA	ACGATGATCTGAGTCCTTGGGCCA	91
***Vcam1***	NM_213891	126 bp	TCCACGCTGGTCATGAATCC	TGGGTCCTTGGGGAAAGAGTA	101

Primers (Integrated DNA technologies, Coralville, IA) that were used for detection of genes were all verified to have reaction efficiency between 90-110% (Table 1). The GeNorm applet v.3.5 (http://medgen.ugent.be/~jvdesomp/genorm/) was used to initially determine the two most stable reference genes from a set of six reference genes using random samples from the cDNA panel generated for the qPCR validation of the microarray. Therefore, normalization of the data was done using the reference genes Hypoxanthine (*Hipox*) and Peptidylprolyl Isomerase A (*Ppia*).

### Statistical analysis

Text files containing the signal and detection *P*-values per probe for each sample were imported into FlexArray software v.1.6.2 (McGill University and Genome Quebec Innovation Centre; http://gqinnovationcenter.com/services/bioinformatics/flexarray/index.aspx?l=e). Data were first processed by analyzing box plot of intensity of samples to ensure that signal intensity data were comparable between samples. Data were then processed by applying a variance-stabilizing normalization (VSN) filter in order to normalize the datasets. The VSN method fits the VSN model to raw microarray data. In contrast with other methods of preprocessing two-color microarray data, VSN normalization is a one-step procedure. The data are returned on a generalized logarithm scale to base 2. A principal component of analysis plot was created to observe separation of different treatment groups. No outliers were removed from the data. Then, scatter plots of expression were analyzed to ensure that probe data within a treatment group were not differentially expressed but also to verify that some probes were differentially expressed between different treatment groups, indicating possible differentially expressed genes. Afterward, an analysis of variance (ANOVA) was used to search for differentially expressed genes between infected and mock-infected group. ANOVA results were then post-processed in flexarray by a FDR correction using a Benjamini Hochberg algorithm. Differentially expressed genes were defined by fold changes greater than 2-folds or smaller than 0.5 fold with an accompanying *P*-value ≤ 0.05. Heat map was then constructed using Cluster 3.0 [43] and Java Tree view 1.1.6r4 [44]. Heat map construction of normalized expression of differentially expressed genes was performed using hierarchical cluster analysis with uncentered correlation used as similarity metric and complete linkage as clustering method.

For RT-qPCR analysis, fold-changes of gene expression were calculated using CFX software manager v.3.0 (Bio-Rad). Samples from mock-infected NPTr cells were used as calibrator. Results were analyzed using Sigmaplot 12.5 (Systat, Chicago, IL), and ANOVA was performed to measure statistical differences between groups. Differences were considered statistically significant at *P* ≤ 0.05.

## Competing interests

All authors declare that they have no competing interests.

## Authors’ contributions

YD, CL, and MG conceived the study. YD, CL, CG, CS, YW, DG and MS have made substantial contributions to the acquisition of the data. All authors read and approved the final version of the manuscript.

## Supplementary Material

Additional file 1 Supplementary Tables. Click here for file
