# Supplementary material for: Transcriptional approach to study porcine tracheal epithelial cells individually or dually infected with swine influenza virus and Streptococcus suis
Source: BMC Vet Res. 2014 Apr 7;10:86. doi: 10.1186/1746-6148-10-86 (PMC4022123; doi:10.1186/1746-6148-10-86)
Supplement: Additional file 1 — Supplementary Tables. [file 1746-6148-10-86-S1.docx]

**Table S1.** List of up-regulated genes greater than two-fold in NPTr cells infected with *S. suis* strain 31533 (B), swine influenza virus H1N1 (V), or co-infected with *S. suis* and H1N1 (V & B). Values represent mean fold increase in infected-NPTr cells compared to mock-infected NPTr cells (*n* = 4 per group).

| **Genebank ID** | **Gene** | **Gene description** | **V & B** | **V** | **B** |
| --- | --- | --- | --- | --- | --- |
| **Cytokines, chemokines, and related receptors** | | | | | |
| NM_214214 | CCL2 | Chemokine (C-C motif) ligand 2 | **57.4** | **49.9** | 1.3 |
| NM_001009579 | CCL3L1 | Chemokine (C-C motif) ligand 3-like 1 | **2.0** | 1.2 | 1.1 |
| NM_213779 | CCL4 | Chemokine (C-C motif) ligand 4 | **146.2** | **110.9** | 1.4 |
| NM_001129946 | CCL5 | Chemokine (C-C motif) ligand 5 | **144.2** | **122.7** | **6.8** |
| NM_001001532 | CCR7 | Chemokine (C-C motif) receptor 7 | **3.7** | **3.3** | 0.9 |
| NM_214118 | CSF2 | Colony stimulating factor 2 (granulocyte-macrophage) | **2.0** | 0.9 | 1.5 |
| NM_001008691 | CXCL10 | Chemokine (C-X-C motif) ligand 10 | **10.7** | **16.1** | 0.6 |
| NM_001128491 | CXCL11 | Chemokine (C-X-C motif) ligand 11 | **6.1** | **7.8** | 1.0 |
| NM_001244128 | CXCL14 | Chemokine (C-X-C motif) ligand 14 | 1.4 | 1.4 | **2.5** |
| NM_001003923 | IFNB1 | Interferon beta | **73.0** | **62.2** | 0.9 |
| NM_213993 | IL12A | Interleukin 12A (p35) | **2.6** | **2.2** | 1.4 |
| NM_213751 | IL16 | Interleukin 16 | 2.0 | 1.4 | **6.8** |
| NM_213997 | IL18 | Interleukin 18 (interferon-gamma-inducing factor) | **2.2** | **2.4** | 1.7 |
| NM_214029 | IL1A | Interleukin 1, alpha | **6.9** | **4.3** | **2.1** |
| NM_001142837 | IFNλ1 | Interferon lambda 1, (Interleukin 29) | **5.5** | **5.4** | 0.8 |
| XM_003481897 | IL4I1 | Interleukin 4 induced 1 | **2.3** | **2.1** | 1.3 |
| NM_214399 | IL6 | Interleukin 6 | **7.9** | **7.5** | 1.1 |
| NM_214135 | IL7 | Interleukin 7 | 1.5 | **2.7** | 0.6 |
| NM_213867 | IL8 | Interleukin 8 | **23.4** | **12.3** | 1.3 |
| NM_214453 | LTA | Lymphotoxin alpha (TNF superfamily, member 1) | **2.5** | **2.5** | 1.3 |
| NM_214295 | NOS3 | Nitric oxide synthase 3 (endothelial cell) | **2.4** | **2.1** | 1.2 |
| NM_213834 | PTGER3 | Prostaglandin E receptor 3 (subtype EP3) | **8.4** | **7.1** | 1.0 |
| NM_214321 | PTGS2 | Prostaglandin-endoperoxide synthase 2 (prostaglandin G/H synthase and cyclooxygenase) | **23.6** | **3.5** | **2.4** |
| NM_214023 | SPP1 | Secreted phosphoprotein 1 | **2.6** | 0.9 | **3.3** |
| NM_214251 | TGFA | Transforming growth factor, alpha | **2.6** | 1.8 | 1.4 |
| NM_214015 | TGFB1 | Transforming growth factor, beta 1 | **2.2** | 1.7 | **2.1** |
| NM_001142839 | TNFRSF12A | Tumor necrosis factor receptor superfamily, member 12A | **4.6** | **3.5** | 1.0 |
| NM_001024696 | TNFSF10 | Tumor necrosis factor (ligand) superfamily, member 10 | **3.2** | **4.9** | 0.9 |
| NM_214084 | VEGFA | Vascular endothelial growth factor A | **3.0** | 1.1 | **2.0** |
| **Cytokine signaling** | | | | | |
| XM_003125501 | ANXA13 | Annexin A13 | **3.7** | **2.5** | **4.4** |
| NM_214034 | CHRM1 | Cholinergic receptor, muscarinic 1 | **4.0** | **4.2** | 1.1 |
| NM_001110428 | CNPY3 | Canopy 3 homolog (zebrafish) | **2.0** | 2.0 | 0.9 |
| NM_213882 | EDN1 | Endothelin 1 | **6.5** | **5.4** | 1.2 |
| NM_001098582 | EDN3 | Endothelin 3 | **2.5** | 1.7 | 1.1 |
| XM_001926976 | GEM | GTP binding protein overexpressed in skeletal muscle | **3.0** | **2.5** | 0.8 |
| NM_001123127 | HSP70 | Heat shock protein 70 | **9.4** | **8.0** | 1.1 |
| NM_213766 | HSP70.2 | Heat shock protein 70.2 | **2.3** | **2.3** | 1.3 |
| XM_003126363 | IRAK3 | Interleukin-1 receptor-associated kinase 3 | **2.2** | 1.6 | 1.9 |
| NM_001097413 | IRF1 | Interferon regulatory factor 1 | **2.5** | **3.1** | 0.9 |
| NM_214278 | IRF6 | Interferon regulatory factor 6 | **2.2** | 1.8 | 1.4 |
| NM_001097428 | IRF7 | Interferon regulatory factor 7 | **9.5** | **12.5** | 1.3 |
| NM_001162401 | LPAR2 | lysophosphatidic acid receptor 2 | **2.7** | **2.7** | 1.1 |
| NM_001038008 | MET | Met proto-oncogene (hepatocyte growth factor receptor) | **3.0** | **2.4** | 1.9 |
| XM_001927183 | NKRF | NFKB repressing factor | **2.4** | 1.9 | 1.5 |
| XM_001928960 | PEA15 | Phosphoprotein enriched in astrocytes 15 | **2.2** | 1.6 | 1.5 |
| NM_214319 | PKR | Double stranded RNA-dependent protein kinase | **5.3** | **6.6** | 1.0 |
| NM_214052 | PLCD4 | Phospholipase C, delta 4 | **2.6** | **2.8** | 1.1 |
| XM_003133981 | PLK2 | Polo-like kinase 2 | **5.0** | **2.3** | **2.2** |
| XM_003128058 | PLK3 | Polo-like kinase 3 | **3.5** | **3.7** | 1.4 |
| NM_214379 | PPARG | Peroxisome proliferator-activated receptor gamma | **2.5** | 1.5 | 1.4 |
| NM_001123185 | RAP2A | RAP2A, member of RAS oncogene family | 1.9 | **2.2** | 1.1 |
| XM_003132454 | RASA2 | RAS p21 protein activator 2 | **2.6** | **2.3** | 1.2 |
| NM_001044600 | RGS2 | Regulator of G-protein signaling 2, 24kDa | **5.8** | **5.9** | 1.5 |
| NM_001185184 | RGS4 | Regulator of G-protein signaling 4 | **5.0** | **2.8** | 1.4 |
| NM_214058 | SERPINA7 | Serpin peptidase inhibitor, clade A (alpha-1 antiproteinase, antitrypsin), member 7 | **2.4** | 1.9 | 1.7 |
| XM_003482119 | SERPINB1 | Serpin peptidase inhibitor, clade B (ovalbumin), member 1 | **2.9** | **2.7** | 1.2 |
| NM_213910 | SERPINE1 | Serpin peptidase inhibitor, clade E (nexin, plasminogen activator inhibitor type 1), member 1 | **4.4** | **3.6** | **3.3** |
| NM_001123196 | SOCS3 | Suppressor of cytokine signaling 3 | 1.6 | **2.2** | 1.1 |
| NM_213769 | STAT1 | Signal transducer and activator of transcription 1, 91kDa | **5.5** | **6.5** | 0.8 |
| NM_213889 | STAT2 | Signal transducer and activator of transcription 2, 113kDa | **2.2** | **2.6** | 0.7 |
| NM_001197305 | STAT4 | Signal transducer and activator of transcription 4 | **2.8** | **3.2** | 1.2 |
| NM_001163649 | TRIM21 | Tripartite motif containing 21 | **3.5** | **3.5** | 1.0 |
| NM_001123209 | TRIM26 | Tripartite motif containing 26 | **2.4** | **2.9** | 0.9 |
| XM_003483413 | UBC | Ubiquitin C | **2.7** | **2.4** | 0.9 |
| NM_001160088 | UBD | Ubiquitin D | **2.1** | **3.0** | 0.6 |
| **Defense response** | | | | | |
| NM_001243703 | ASB9 | Ankyrin repeat and SOCS box containing 9 | **11.0** | **14.9** | 0.7 |
| NM_001161755 | BST2 | Bone marrow stromal cell antigen 2 | **10.6** | **13.9** | 1.2 |
| XM_003126517 | C1R | Complement component 1, r subcomponent | **2.7** | **4.5** | 0.7 |
| NM_001005349 | C1S | Complement component 1, s subcomponent | **4.2** | **9.1** | 1.2 |
| NM_001123089 | C4 | Complement C4 | **2.1** | 0.8 | 0.8 |
| NM_213942 | C4BPA | Complement component 4 binding protein, alpha | **2.6** | **3.8** | 0.9 |
| NM_214281 | CFH | Complement factor H | **2.3** | **4.0** | 0.8 |
| XM_003483530 | CYP26A1 | Cytochrome P450, family 26, subfamily A, polypeptide 1 | **3.8** | 1.3 | 1.5 |
| XM_001927604 | DDX18 | DEAD (Asp-Glu-Ala-Asp) box polypeptide 18 | **2.7** | **2.7** | 1.5 |
| XM_001928772 | DDX21 | DEAD (Asp-Glu-Ala-Asp) box polypeptide 21 | **2.4** | **2.2** | 1.8 |
| NM_213804 | DDX58 | DEAD (Asp-Glu-Ala-Asp) box polypeptide 58 | **33.6** | **37.1** | 1.1 |
| NM_001199132 | DHX58 | DEXH (Asp-Glu-X-His) box polypeptide 58 | **27.4** | **34.8** | 1.0 |
| NM_001033011 | FCGR1A | Fc fragment of IgG, high affinity Ia, receptor (CD64) | **2.6** | **2.9** | 0.9 |
| NM_001033013 | FCGR2B | Fc fragment of IgG, low affinity IIb, receptor (CD32) | **2.5** | **2.4** | 1.0 |
| NM_214391 | FCGR3B | Fc fragment of IgG, low affinity IIIb, receptor (CD16b) | **5.1** | **8.2** | 0.8 |
| NM_214391 | GBP1 | Guanylate binding protein 1, interferon-inducible | **21.6** | **26.9** | 1.0 |
| NM_001128474 | GBP2 | Guanylate binding protein 2, interferon-inducible | **7.2** | **8.9** | 0.9 |
| XM_001927929 | GBP4 | Guanylate binding protein 4 | **29.3** | **42.2** | 1.0 |
| NM_214117 | HAMP | Hepcidin antimicrobial peptide | **4.3** | **3.9** | 1.2 |
| NM_214299 | HBEGF | Heparin-binding EGF-like growth factor | **4.2** | **2.3** | 1.4 |
| XM_001929212 | IFI16 | Interferon activated gene 203 | **10.6** | **16.4** | 0.8 |
| NM_001246205 | IFI44 | Interferon-induced protein 44 | **15.6** | **16.4** | 0.9 |
| NM_001100194 | IFIH1 | Interferon induced with helicase C domain 1 | **11.1** | **13.4** | 0.8 |
| NM_001244363 | IFIT1 | Interferon-induced protein with tetratricopeptide repeats 1 | **113.8** | **146.4** | 1.0 |
| XM_001928671 | IFIT2 | Interferon-induced protein with tetratricopeptide repeats 2 | **112.3** | **151.2** | 0.8 |
| NM_001204395 | IFIT3 | Interferon-induced protein with tetratricopeptide repeats 3 | **185.5** | **170.0** | 1.6 |
| XM_001925952 | IFIT5 | Interferon-induced protein with tetratricopeptide repeats 5 | **3.5** | **4.2** | 0.9 |
| XM_003124230 | IFITM1 | Interferon induced transmembrane protein 1 (9-27) | **6.0** | **6.6** | 0.9 |
| NM_001007519 | IFRD1 | Interferon-related developmental regulator 1 | **4.2** | **3.2** | 1.8 |
| NM_213817 | IRG6 | Inflammatory response protein 6 | **272.3** | **353.7** | 1.1 |
| NM_001198921 | ISG12(A) | Putative ISG12(a) protein | **3.4** | **3.6** | 0.9 |
| NM_001128469 | ISG15 | ISG15 ubiquitin-like modifier | **102.6** | **100.6** | **2.3** |
| NM_001105306 | LAG3 | Lymphocyte-activation gene 3 | **4.1** | **2.7** | 0.9 |
| NM_001122984 | MFGE8 | Milk fat globule-EGF factor 8 protein | **2.1** | 1.0 | 1.7 |
| NM_214061 | MX1 | Myxovirus (influenza virus) resistance 1, interferon-inducible protein p78 (mouse) | **73.8** | **81.9** | 1.3 |
| NM_001097416 | MX2 | Myxovirus (influenza virus) resistance 2 (mouse) | **57.9** | **70.6** | 1.3 |
| NM_214303 | OAS1 | 2'-5'-oligoadenylate synthetase 1, 40/46kDa | **78.6** | **88.7** | 1.1 |
| NM_001031796 | OAS2 | 2'-5'-oligoadenylate synthetase 2, 69/71kDa | **29.0** | **34.2** | 0.9 |
| NM_001031790 | OASL | 2'-5'-oligoadenylate synthetase-like | **84.3** | **130.6** | 1.2 |
| NM_213805 | OLR1 | Oxidized low density lipoprotein (lectin-like) receptor 1 | **3.9** | **7.4** | 0.1 |
| NM_001244503 | PIK3AP1 | Phosphoinositide-3-kinase adaptor protein 1 | **3.2** | **2.6** | 1.4 |
| NM_001144901 | PSMA5 | Proteasome (prosome, macropain) subunit, alpha type, 5 | 1.9 | **2.1** | 1.3 |
| NM_001044565 | PSMB10 | Proteasome (prosome, macropain) subunit, beta type, 10 | **2.3** | **2.5** | 1.2 |
| NM_213935 | PSMB8 | Proteasome (prosome, macropain) subunit, beta type, 8 (large multifunctional peptidase 7) | **2.7** | **3.0** | 1.0 |
| NM_001037961 | PSMB9 | Proteasome (prosome, macropain) subunit, beta type, 9 (large multifunctional peptidase 2) | **7.6** | **9.0** | 1.2 |
| NM_214279 | PSME2 | Proteasome (prosome, macropain) activator subunit 2 (PA28 beta) | **2.5** | **2.5** | 1.1 |
| XM_003130143 | SAMD9 | Sterile alpha motif domain containing 9 | **6.8** | **8.0** | 1.0 |
| XM_003121524 | SEMA6D | Sema domain, transmembrane domain (TM), and cytoplasmic domain, (semaphorin) 6D | **2.8** | 0.9 | 1.2 |
| XM_001929523 | SH2B3 | SH2B adaptor protein 3 | **3.4** | **3.3** | 1.2 |
| NM_001114064 | SLA-DOB | MHC class II, DO beta | **3.2** | **2.3** | 1.1 |
| NM_001044581 | TAP1 | Transporter 1, ATP-binding cassette, sub-family B (MDR/TAP) | **5.2** | **5.2** | 0.9 |
| NM_001206441 | TAP2 | Transporter 2, ATP-binding cassette, sub-family B (MDR/TAP) | **3.3** | **3.8** | 1.2 |
| **Transcriptional and translational regulation** | | | | | |
| NM_001123078 | ATF4 | Activating transcription factor 4 (tax-responsive enhancer element B67) | **2.5** | **2.1** | 1.9 |
| XM_001928628 | CDCA7 | Cell division cycle associated 7 | **3.1** | **3.0** | **2.5** |
| NM_001097510 | CPEB1 | Cytoplasmic polyadenylation element binding protein 1 | **2.5** | **2.3** | 1.0 |
| NM_213991 | DNASE1 | Deoxyribonuclease I | **2.0** | 1.9 | 1.5 |
| NM_001243674 | DNTTIP2 | Deoxynucleotidyltransferase, terminal, interacting protein 2 | **2.2** | 1.7 | 1.2 |
| NM_001243545 | EEF1E1 | Eukaryotic translation elongation factor 1 epsilon 1 | **2.2** | 1.9 | 1.5 |
| NM_001194975 | EIF2C2 | Eukaryotic translation initiation factor 2C, 2 | **2.4** | 1.5 | 1.8 |
| NM_001100196 | EIF4A1 | Eukaryotic translation initiation factor 4A1 | **2.1** | **2.0** | 1.3 |
| XM_003123701 | ENC1 | Ectodermal-neural cortex 1 (with BTB-like domain) | **2.2** | 1.2 | 1.1 |
| NM_001162886 | ETS1 | V-ets erythroblastosis virus E26 oncogene homolog 1 (avian) | **2.4** | **2.1** | 1.7 |
| XM_001929006 | EXOSC3 | Exosome component 3 | **2.1** | 1.9 | 1.4 |
| NM_001044567 | GATA3 | GATA binding protein 3 | **2.8** | **3.3** | 0.9 |
| NM_001185155 | HMGA1 | High mobility group AT-hook 1 | **2.5** | **2.5** | 1.6 |
| NM_001243292 | IMP3 | IMP3, U3 small nucleolar ribonucleoprotein, homolog (yeast) | **2.1** | 1.7 | 1.5 |
| NM_001134344 | KLF10 | Kruppel-like factor 10 | **2.1** | 1.3 | 1.1 |
| NM_001164009 | KLF16 | Kruppel-like factor 16 | **2.3** | 1.6 | 1.7 |
| NM_001031782 | KLF4 | Kruppel-like factor 4 (gut) | **3.0** | **2.8** | 1.3 |
| NM_001134353 | KLF6 | Kruppel-like factor 6 | **4.1** | 1.7 | **2.3** |
| XM_003121764 | LARP6 | La ribonucleoprotein domain family, member 6 | **2.4** | 1.5 | 1.4 |
| XM_003132764 | MORC3 | MORC family CW-type zinc finger 3 | **2.0** | **2.3** | 0.7 |
| XM_001929271 | MTERFD1 | MTERF domain containing 1 | **2.0** | 1.6 | 1.3 |
| NM_001005154 | MYC | V-myc myelocytomatosis viral oncogene homolog (avian) | **4.4** | **2.7** | 1.6 |
| NM_001012406 | MYOG | Myogenin (myogenic factor 4) | 2.0 | **2.1** | 0.9 |
| XM_003134615 | PARP12 | Poly (ADP-ribose) polymerase family, member 12 | **16.6** | **18.0** | 1.0 |
| XM_003483310 | PARP14 | Poly (ADP-ribose) polymerase family, member 14 | **7.3** | **11.4** | 0.8 |
| XM_003129196 | PHF17 | PHD finger protein 17 | **2.3** | 1.7 | 1.4 |
| XM_001927933 | POLR3D | Polymerase (RNA) III (DNA directed) polypeptide D, 44kDa | **2.9** | **2.2** | 1.6 |
| NM_001044564 | SFN | Stratifin | **2.4** | 1.9 | 1.7 |
| XM_001927743 | SNAPC3 | Small nuclear RNA activating complex, polypeptide 3, 50kDa | **2.4** | 1.9 | 1.3 |
| XM_003127875 | TAF4B | TAF4b RNA polymerase II, TATA box binding protein (TBP)-associated factor, 105kDa | **2.6** | 1.4 | 1.1 |
| XM_001924680 | TDRD7 | Tudor domain containing 7 | **3.6** | **3.4** | 0.9 |
| NM_001142666 | TEAD4 | TEA domain family member 4 | **2.2** | 1.9 | 1.4 |
| XM_003484256 | TTF2 | Transcription termination factor, RNA polymerase II | **2.1** | **2.3** | 1.2 |
| XM_001929654 | VPS72 | Vacuolar protein sorting 72 homolog (S. cerevisiae) | **2.1** | **2.1** | 1.7 |
| XM_003121357 | ZBTB24 | Zinc finger and BTB domain containing 24 | **2.1** | 1.7 | 1.2 |
| XM_003124590 | ZC3H7A | Zinc finger CCCH-type containing 7A | **2.0** | 1.5 | 1.1 |
| **Apoptosis, cell cycle regulation, and oncogenesis** | | | | | |
| NM_214454 | AQP1 | Aquaporin 1 (Colton blood group) | **2.1** | 0.5 | **2.3** |
| NM_214376 | AREG | Amphiregulin | **3.5** | 1.9 | 1.3 |
| NM_001128458 | AXL | AXL receptor tyrosine kinase | **2.6** | 1.8 | 1.3 |
| NM_001030535 | BID | BH3 interacting domain death agonist | **2.2** | **2.8** | 1.4 |
| XM_003358029 | BRCA1 | Breast cancer 1, early onset | 1.6 | **2.0** | 1.0 |
| NM_001161640 | CASP10 | Caspase 10, apoptosis-related cysteine peptidase | **3.0** | **3.2** | 1.5 |
| XM_001927446 | CCDC60 | Coiled-coil domain containing 60 | **2.1** | 2.0 | 1.4 |
| NM_001078678 | CCND3 | Cyclin D3 | **2.0** | 1.1 | 1.2 |
| XM_001928033 | CCNJ | Cyclin J | **2.0** | 1.6 | 1.3 |
| NM_001129970 | CYCS | Cytochrome c, somatic | **2.2** | 1.5 | 1.4 |
| NM_001044599 | GADD45A | Growth arrest and DNA-damage-inducible, alpha | **2.7** | 1.8 | 1.4 |
| NM_001185129 | GADD45G | Growth arrest and DNA-damage-inducible, gamma | **3.1** | 2.0 | 1.4 |
| XM_001928454 | GNL3 | Guanine nucleotide binding protein-like 3 (nucleolar) | **2.2** | **2.1** | 1.7 |
| NM_001005156 | IGFBP3 | Insulin-like growth factor binding protein 3 | **2.0** | 1.3 | **2.3** |
| NM_214361 | MCL1 | Myeloid cell leukemia sequence 1 (BCL2-related) | **2.9** | 1.4 | 1.1 |
| NM_001105303 | MDM2 | Mdm2 p53 binding protein homolog (mouse) | **2.1** | **2.3** | 1.2 |
| NM_214147 | PMAIP1 | Phorbol-12-myristate-13-acetate-induced protein 1 | **5.3** | **3.9** | 1.5 |
| XM_001925572 | PML | Promyelocytic leukemia | **3.7** | **3.9** | 1.0 |
| XM_003128852 | PNAS-5 | Apoptosis-related protein | **3.4** | **3.3** | **2.0** |
| XM_001929178 | WDR3 | WD repeat domain 3 | 1.8 | **2.0** | 1.5 |
| **Cell adhesion and migration** | | | | | |
| XM_001925781 | ADAM8 | ADAM metallopeptidase domain 8 | **5.1** | 2.0 | **2.9** |
| NM_001161645 | CLDN6 | Claudin 6 | **2.8** | **2.2** | 1.0 |
| NM_001097519 | GJD3 | Gap junction protein, delta 3, 31.9kDa | **2.1** | 1.4 | 1.4 |
| NM_001244272 | ITGA2 | Integrin, alpha 2 (CD49B, alpha 2 subunit of VLA-2 receptor) | **2.0** | 1.3 | 1.5 |
| NM_213968 | ITGB1 | Integrin, beta 1 (fibronectin receptor, beta polypeptide, antigen CD29 includes MDF2, MSK12) | **2.1** | 1.8 | 1.3 |
| NM_001142827 | LGALS8 | Lectin, galactoside-binding, soluble, 8 | **2.7** | **2.6** | 1.1 |
| NM_213932 | LGALS9 | Lectin, galactoside-binding, soluble, 9 | **13.7** | **15.2** | 1.9 |
| XM_003129808 | MMP-13 | Matrix metalloproteinase 13 precursor | **2.0** | 1.4 | 1.3 |
|  | PCDH18 | Protocadherin 18 | **3.1** | **3.0** | 1.6 |
| NM_214284 | SDC4 | Syndecan 4 | **2.2** | 1.9 | 1.4 |
| NM_001244536 | THBS1 | Thrombospondin 1 | **2.4** | 1.5 | 1.1 |
| NM_213891 | VCAM1 | Vascular cell adhesion molecule 1 | **3.9** | **2.7** | 0.8 |
| NM_213934 | VCL | Vinculin | **2.0** | 1.4 | 1.2 |
| **Surface receptor molecule, antigen presentation and co-stimulation** | | | | | |
| NM_001037148 | ADRA2B | Adrenergic, alpha-2B-, receptor | 2.0 | **2.1** | 1.1 |
| NM_001244800 | CD5 | CD5 molecule | **3.0** | **2.6** | 0.9 |
| NM_001135962 | CD79A | CD79a molecule, immunoglobulin-associated alpha | **3.3** | **2.4** | 1.3 |
| NM_214222 | CD86 | CD86 molecule | 1.8 | **2.0** | 0.9 |
| XM_001926883 | MUC1 | Mucin 1, cell surface associated | **2.5** | 1.5 | **2.4** |
| NM_214001 | TFRC | Transferrin receptor (p90, CD71) | **2.3** | 1.8 | **2.2** |
| **Angiogenesis** | | | | | |
| NM_001038644 | ANGPTL4 | Angiopoietin-like 4 | **2.3** | 1.9 | **2.6** |
| XM_001927740 | CYR61 | Cysteine-rich, angiogenic inducer, 61 | **6.7** | **2.4** | 1.6 |
| NM_001122985 | F2 | Coagulation factor II (thrombin) | **2.2** | 1.8 | 1.5 |
| XM_001926867 | PTPRB | Protein tyrosine phosphatase, receptor type, B | **2.2** | 1.3 | 1.5 |
| **Cytoskeleton/actin rearrangement** | | | | | |
| NM_001167795 | ACTA1 | Actin, alpha 1, skeletal muscle | **4.6** | **3.5** | 1.5 |
| NM_001164650 | ACTA2 | Actin, alpha 2, smooth muscle, aorta | **2.4** | 0.8 | 0.8 |
| NM_001170517 | ACTC1 | Actin, alpha, cardiac muscle 1 | **2.1** | 0.3 | 0.5 |
| NM_001204181 | AKAP2 | A kinase (PRKA) anchor protein 2 | **4.1** | **3.3** | 1.7 |
| NM_001190193 | CDC42SE1 | CDC42 small effector 1 | **2.4** | **2.2** | 1.4 |
| XM_001928351 | CEP55 | Centrosomal protein 55kDa | **3.0** | **2.3** | 1.3 |
| NM_213878 | CNN1 | Calponin 1, basic, smooth muscle | **2.3** | 1.4 | 1.7 |
| XM_003125902 | CNN3 | Calponin 3, acidic | **2.9** | **2.2** | 1.8 |
| XM_001924233 | EZR | Ezrin | **2.0** | 1.8 | 1.3 |
| NM_001123141 | MYH4 | Myosin, heavy chain 4, skeletal muscle | **2.2** | **2.6** | 1.1 |
| XM_003131994 | MYHC | Myosin heavy chain | **2.9** | **2.5** | 0.8 |
| XM_001928969 | NUP43 | Nucleoporin 43kDa | 1.9 | **2.2** | 1.4 |
| NM_001243427 | TCP1 | T-complex 1 | 1.9 | **2.1** | 1.0 |
| NM_001243294 | TMOD4 | Tropomodulin 4 (muscle) | **4.0** | **3.4** | 1.1 |
| NM_001044612 | TUBB2A | Tubulin, beta 2A class IIa | **2.1** | **2.2** | 1.3 |
| **Lipid metabolism** | | | | | |
| NM_214388 | APOA4 | Apolipoprotein A-IV | 1.4 | **2.3** | 1.2 |
| XM_003127332 | CPT1C | Carnitine palmitoyltransferase 1C | **3.5** | **3.4** | 1.1 |
| NM_001039746 | FABP5 | Fatty acid binding protein 5 (psoriasis-associated) | **2.9** | **2.5** | 1.7 |
| NM_001206354 | LDLR | Low density lipoprotein receptor | **2.7** | 1.7 | **2.4** |
| NM_001199891 | LRP8 | Low density lipoprotein receptor-related protein 8, apolipoprotein e receptor | **2.2** | 1.9 | **2.0** |
| NM_001143718 | MGLL | Monoglyceride lipase | **2.4** | **2.0** | **2.0** |
| NM_001204766 | O3FAR1 | Omega-3 fatty acid receptor 1 | **2.1** | 1.4 | 1.5 |
| NM_213739 | PAQR7 | Progestin and adipoQ receptor family member VII | **3.2** | 1.9 | 1.5 |
| XM_001928435 | PLEK2 | Pleckstrin 2 | **2.1** | **2.2** | 1.5 |
| NM_213967 | SCARB1 | Scavenger receptor class B, member 1 | **2.0** | 1.6 | 1.7 |
| **Biological and metabolic processes** | | | | | |
| XM_001925989 | ABHD2 | Abhydrolase domain containing 2 | 2.0 | **2.1** | 1.5 |
| NM_001097508 | ADSS | Adenylosuccinate synthase | **2.1** | **2.0** | 1.3 |
| NM_214310 | AMH | Anti-Mullerian hormone | **2.6** | **2.8** | 1.0 |
| NM_001123076 | AMPD1 | Adenosine monophosphate deaminase 1 | **3.6** | **3.1** | 0.9 |
| XM_001928460 | ANKRD22 | Ankyrin repeat domain 22 | **2.7** | **2.4** | 1.2 |
| NM_001078677 | ARF6 | ADP-ribosylation factor 6 | **3.4** | **2.5** | 1.9 |
| XM_003480246 | ARG1 | Arginase, liver | **2.4** | 1.6 | 1.2 |
| XM_001928060 | ARHGAP30 | Rho GTPase activating protein 30 | **3.7** | **3.6** | 1.0 |
| NM_001244315 | ARL4C | ADP-ribosylation factor-like 4C | **3.8** | 1.7 | **2.2** |
| XM_001926554 | ASPHD2 | Aspartate beta-hydroxylase domain containing 2 | **2.2** | **2.3** | 1.2 |
| XM_001927977 | ATAD1 | ATPase family, AAA domain containing 1 | **2.4** | **2.3** | 1.0 |
| NM_001128488 | AZIN1 | Antizyme inhibitor 1 | **2.0** | 1.5 | 1.3 |
| NM_213978 | B2M | Beta-2-microglobulin | **2.3** | **2.3** | 1.0 |
| NM_214351 | B3GALNT1 | Beta-1,3-N-acetylgalactosaminyltransferase 1 (globoside blood group) | **2.1** | 1.5 | 1.5 |
| XM_001925895 | BCR | Breakpoint cluster region | **2.5** | **2.8** | 1.1 |
| NM_214259 | BDNF | Brain-derived neurotrophic factor | **2.1** | 1.5 | 0.9 |
| NM_001195399 | BMP2 | Bone morphogenetic protein 2 | **2.2** | **2.9** | 1.3 |
| XM_001928781 | BMS1 | BMS1 homolog, ribosome assembly protein (yeast) | **2.1** | **2.0** | 1.3 |
| NM_213846 | BNP | Brain natriuretic peptide | **5.0** | **2.6** | 1.2 |
| NM_001243920 | CA4 | Carbonic anhydrase IV | 1.9 | **2.6** | 1.1 |
| NM_213922 | CARP | Cardiac ankyrin repeat protein | **2.8** | 0.4 | 0.6 |
| NM_001123092 | CBLN4 | Cerebellin 4 precursor | **2.0** | **2.0** | 1.3 |
| NM_213833 | CCN2 | Connective tissue growth factor | **5.2** | **2.7** | 1.4 |
| NM_214081 | CHGB | Chromogranin B (secretogranin 1) | **2.4** | **2.6** | 1.2 |
| NM_001244442 | CHSY1 | Chondroitin sulfate synthase 1 | **3.0** | **2.7** | 1.9 |
| NM_001164648 | CILP | Cartilage intermediate layer protein, nucleotide pyrophosphohydrolase | 1.1 | **2.0** | 1.1 |
| NM_001004026 | CSN3 | Casein kappa | **3.4** | **2.5** | 1.0 |
| NM_001144845 | DDIT3 | DNA-damage-inducible transcript 3 | 1.9 | **2.5** | 1.0 |
| NM_001142668 | DKC1 | Dyskeratosis congenita 1, dyskerin | **2.2** | **2.3** | 1.8 |
| XM_003121929 | DOCK8 | Dedicator of cytokinesis 8 | **2.2** | 2.0 | 0.9 |
| XM_001926423 | DSCC1 | Defective in sister chromatid cohesion 1 homolog (S. cerevisiae) | **2.8** | **2.6** | 1.5 |
| XM_003480399 | EHD4 | EH-domain containing 4 | **2.1** | 1.8 | 1.5 |
| NM_001099940 | EMP1 | Epithelial membrane protein 1 | **3.8** | **2.6** | **2.2** |
| NM_214134 | EPO | Erythropoietin | 1.2 | **2.1** | 0.8 |
| NM_001137627 | ERO1L | ERO1-like (S. cerevisiae) | 1.3 | 0.9 | **2.3** |
| XM_001928323 | FRMD6 | FERM domain containing 6 | **2.1** | 1.2 | 1.3 |
| XM_001926014 | FRMPD1 | FERM and PDZ domain containing 1 | **3.5** | 1.3 | **2.9** |
| NM_001003662 | FST | Follistatin | **3.1** | **2.6** | 1.1 |
| NM_213899 | FUT8 | Fucosyltransferase 8 (alpha (1,6) fucosyltransferase) | **2.2** | 2.0 | 1.3 |
| NM_213807 | GHRL | Ghrelin/obestatin prepropeptide | **2.7** | **3.0** | 1.1 |
| XM_001929120 | GMPR | Guanosine monophosphate reductase | **2.3** | **3.8** | 0.9 |
| NM_001243376 | GNPNAT1 | Glucosamine-phosphate N-acetyltransferase 1 | **2.2** | 1.5 | 1.1 |
| XM_003134242 | GPCPD1 | Glycerophosphocholine phosphodiesterase GDE1 homolog (S. cerevisiae) | 1.9 | **2.0** | 1.3 |
| NM_001160275 | GUCA2B | Guanylate cyclase activator 2B (uroguanylin) | **3.6** | **3.6** | 1.1 |
| NM_001001268 | HAS3 | Hyaluronan synthase 3 | **6.2** | 1.6 | 1.9 |
| NM_001122987 | HK2 | Hexokinase 2 | **3.2** | 1.5 | **2.7** |
| NM_001122988 | HMGCR | 3-hydroxy-3-methylglutaryl-CoA reductase | 1.3 | 0.8 | **2.1** |
| NM_001004027 | HMOX1 | Heme oxygenase (decycling) 1 | **2.3** | **2.1** | 0.9 |
| NM_001112695 | HNRNPAB | Heterogeneous nuclear ribonucleoprotein A/B | **2.1** | 1.9 | 1.4 |
| NM_001136511 | HUS1 | HUS1 checkpoint homolog (S. pombe) | **3.0** | **2.4** | 1.8 |
| XM_001927338 | IDH3A | Isocitrate dehydrogenase 3 (NAD+) alpha | **2.3** | 1.9 | 1.6 |
| NM_213985 | KCNN3 | Potassium intermediate/small conductance calcium-activated channel, subfamily N, member 3 | **2.3** | 0.7 | 1.6 |
| NM_214018 | KCNN4 | Potassium intermediate/small conductance calcium-activated channel, subfamily N, member 4 | **2.7** | **2.1** | 1.8 |
| XM_003126180 | KRT18 | Keratin 18 | **3.8** | **2.8** | **3.0** |
| XM_003126173 | KRT5 | Keratin 5 | **2.2** | 1.0 | **2.8** |
| NM_001159615 | KRT8 | Keratin 8 | **2.1** | 1.5 | 1.8 |
| NM_001244951 | LRRC59 | Leucine rich repeat containing 59 | **2.7** | **3.0** | 1.8 |
| XM_003353358 | LYSMD2 | LysM, putative peptidoglycan-binding, domain containing 2 | **2.1** | 1.7 | 1.4 |
| NM_001128471 | MEST | Mesoderm specific transcript homolog (mouse) | **3.1** | **2.2** | **2.9** |
| XM_003127828 | MFSD2A | Major facilitator superfamily domain containing 2A | **7.1** | **4.7** | **2.6** |
| NM_214116 | MGP | Matrix Gla protein | 1.5 | 1.2 | **2.8** |
| NM_001244607 | MLLT11 | Myeloid/lymphoid or mixed-lineage leukemia (trithorax homolog, Drosophila); translocated to, 11 | **4.3** | **3.3** | 1.5 |
| NM_001001266 | MT1A | Metallothionein 1A | **4.2** | **3.1** | **2.4** |
| XM_001929078 | MYLK | Myosin light chain kinase | **2.1** | 1.1 | 1.6 |
| NM_001031793 | NAMPT | Nicotinamide phosphoribosyltransferase | **3.5** | **3.2** | 1.5 |
| XM_003130607 | NAV1 | Neuron navigator 1 | **2.3** | 1.9 | 1.8 |
|  | ND6 | NADH dehydrogenase subunit 6 | 1.5 | **3.5** | 0.9 |
| NM_001167652 | NDUFAB1 | NADH dehydrogenase (ubiquinone) 1, alpha/beta subcomplex, 1, 8kDa | **3.1** | **2.8** | 1.0 |
| NM_001048071 | NIP7 | Nuclear import 7 homolog (S. cerevisiae) | **2.7** | **2.5** | 1.5 |
| NM_001123145 | NMB | Neuromedin B | **2.6** | 1.8 | 1.1 |
| XM_001928616 | NOC4L | Nucleolar complex associated 4 homolog (S. cerevisiae) | **2.1** | **2.2** | 1.8 |
| NM_001244246 | NOP2 | NOP2 nucleolar protein homolog (yeast) | **2.6** | **2.5** | 1.9 |
| NM_001244305 | NOP56 | NOP56 ribonucleoprotein homolog (yeast) | **2.5** | **2.5** | 1.8 |
| XM_003483715 | NOP58 | NOP58 ribonucleoprotein homolog (yeast) | 1.8 | **2.1** | 1.5 |
| NM_001195362 | NPM2 | Nucleophosmin/nucleoplasmin 2 | **3.8** | **3.4** | 1.1 |
| NM_001244491 | OBFC2A | Oligonucleotide/oligosaccharide-binding fold containing 2A | **4.5** | **2.9** | 1.5 |
| XM_003355281 | OLFM3 | Olfactomedin 3 | **2.1** | 1.7 | 1.2 |
| NM_001098597 | OSTN | Osteocrin | 1.6 | 0.8 | **2.1** |
| NM_214070 | OVGP1 | Oviductal glycoprotein 1, 120kDa | **2.5** | 1.7 | 1.1 |
| NM_001243677 | P2RY10 | Purinergic receptor P2Y, G-protein coupled, 10 | 1.2 | **2.1** | 1.2 |
| NM_001006591 | P2RY2 | Purinergic receptor P2Y, G-protein coupled, 2 | **4.1** | **2.6** | **2.5** |
| NM_001123160 | PFDN4 | Prefoldin subunit 4 | **2.5** | **2.1** | 0.8 |
| NM_001134968 | PGAM2 | Phosphoglycerate mutase 2 (muscle) | **2.5** | 1.4 | **2.1** |
| XM_001925764 | PGF | Placenta growth factor | **2.1** | **2.3** | 1.1 |
| NM_214115 | PHI-1 | Ubiquitous PKC-potentiated PP1 inhibitor | **2.2** | **2.1** | 1.8 |
| NM_214054 | PLAT | Plasminogen activator, tissue | **2.7** | **2.1** | **2.2** |
| NM_213945 | PLAU | Plasminogen activator, urokinase | **20.2** | **8.6** | **2.1** |
| NM_214287 | PN-1 | Nexin-1 | **2.5** | 1.9 | 1.7 |
| XM_001929223 | PNP | Purine nucleoside phosphorylase | **4.3** | **3.0** | **2.7** |
| NM_001244259 | POGK | Pogo transposable element with KRAB domain | **2.0** | 1.6 | 1.4 |
| NM_001128480 | PPP1R18 | Protein phosphatase 1, regulatory subunit 18 | **2.0** | 1.9 | 1.2 |
| XM_003483529 | PPP1R3C | Protein phosphatase 1, regulatory subunit 3C | **5.9** | **4.9** | **3.0** |
| NM_001246670 | PRLHR | Prolactin releasing hormone receptor | **2.3** | **2.0** | 0.9 |
| NM_001008687 | PRNP | Prion protein | **2.2** | 1.7 | 1.5 |
| NM_213916 | PTHLH | Parathyroid hormone-like hormone | **8.1** | **2.5** | 1.9 |
| XM_003132854 | PUS1 | Pseudouridylate synthase 1 | **2.3** | **2.2** | 1.8 |
| XM_003122588 | PYGM | Phosphorylase, glycogen, muscle | **5.1** | **4.4** | 0.9 |
| XM_003132668 | QTRTD1 | Queuine tRNA-ribosyltransferase domain containing 1 | **2.2** | **2.0** | 1.2 |
| XM_003356619 | RAB44 | RAB44, member RAS oncogene family | **2.6** | **2.4** | 1.1 |
| XM_001925433 | RAN | RAN, member RAS oncogene family | **2.0** | **2.1** | 1.4 |
| NM_001185070 | RANBP1 | RAN binding protein 1 | 2.0 | **2.1** | 1.5 |
| NM_001243913 | RCAN1 | Regulator of calcineurin 1 | **2.3** | 1.3 | 1.1 |
| NM_001097512 | RNASEL | Ribonuclease L (2',5'-oligoisoadenylate synthetase-dependent) | 1.6 | **2.2** | 0.7 |
| XM_003480609 | RPL12 | Ribosomal protein L12 | **2.3** | 1.1 | 1.1 |
| NM_213764 | RPS23 | Ribosomal protein S23 | **3.1** | **2.1** | 1.1 |
| NM_001142834 | RSF1 | Remodeling and spacing factor 1 | **3.6** | **2.9** | 1.1 |
| NM_001190168 | S100A14 | S100 calcium binding protein A14 | **3.2** | **3.6** | 1.1 |
| NM_214140 | S100G | S100 calcium binding protein G | **4.0** | 0.9 | **11.0** |
| NM_214358 | SAT1 | Spermidine/spermine N1-acetyltransferase 1 | **3.1** | **2.3** | 0.5 |
| NM_001012299 | SCG2 | Secretogranin II | **6.0** | **4.4** | **2.0** |
| XM_001928302 | SDS | Serine dehydratase | **5.4** | **7.1** | 1.1 |
| NM_001243594 | SELRC1 | Sel1 repeat containing 1 | **2.1** | 2.0 | 1.6 |
| NM_001044533 | SFTPC | Surfactant protein C | **2.2** | 1.7 | 0.9 |
| NM_214178 | SLC23A2 | Solute carrier family 23 (nucleobase transporters), member 2 | **2.4** | **2.2** | 1.4 |
| NM_001164510 | SLC25A25 | Solute carrier family 25 (mitochondrial carrier; phosphate carrier), member 25 | **2.3** | **2.3** | 1.5 |
| XM_003128425 | SLC29A1 | Solute carrier family 29 (nucleoside transporters), member 1 | **2.2** | **2.3** | 1.0 |
| XM_003482114 | SLC2A1 | Solute carrier family 2 (facilitated glucose transporter), member 1 | 1.5 | 0.8 | **2.1** |
| NM_001243667 | SLC41A1 | Solute carrier family 41, member 1 | **2.2** | 1.9 | 1.5 |
| NM_001012613 | SLC7A1 | Solute carrier family 7 (cationic amino acid transporter, y+ system), member 1 | **2.4** | 2.0 | 1.5 |
| XM_003360551 | SLC7A11 | Solute carrier family 7 (anionic amino acid transporter light chain, xc- system), member 11 | 1.7 | **2.3** | 1.1 |
| NM_001110420 | SLC7A2 | Solute carrier family 7 (cationic amino acid transporter, y+ system), member 2 | 2.0 | **2.1** | 1.8 |
| NM_001113697 | SLMO2 | Slowmo homolog 2 (Drosophila) | **3.1** | **2.2** | 1.3 |
| XM_001924930 | SNX31 | Sorting nexin 31 | **3.4** | **3.8** | 1.2 |
| NM_213843 | SOX9 | SRY (sex determining region Y)-box 9 | **2.3** | **2.1** | 1.7 |
| NM_001031776 | SPMI | Seminal plasma sperm motility inhibitor/spermadhesin AQN-3-like protein | **5.1** | 1.2 | 1.0 |
| NM_001101026 | SQLE | Squalene epoxidase | 1.4 | 0.7 | **2.3** |
| XM_003360571 | TCN1 | Transcobalamin I (vitamin B12 binding protein, R binder family) | **4.9** | **3.7** | 1.2 |
|  | TECPR2 | Tectonin beta-propeller repeat containing 2 | **2.8** | **2.9** | 1.0 |
| XM_003127752 | TINAGL1 | Tubulointerstitial nephritis antigen-like 1 | **2.3** | 1.6 | 1.6 |
| XM_003358627 | TIPARP | TCDD-inducible poly(ADP-ribose) polymerase | **2.3** | 1.7 | 1.5 |
| XM_001926935 | TOMM20 | Translocase of outer mitochondrial membrane 20 homolog (yeast) | **3.0** | **2.5** | 1.8 |
| NM_001077221 | TRA2B | Transformer 2 beta homolog (Drosophila) | **3.6** | **2.1** | **2.1** |
| XM_001928643 | UAP1 | UDP-N-acteylglucosamine pyrophosphorylase 1 | **2.8** | **2.1** | 1.6 |
| XM_001927934 | URB2 | URB2 ribosome biogenesis 2 homolog (S. cerevisiae) | **2.4** | **2.4** | 1.8 |
| XM_003481645 | USP15 | Ubiquitin specific peptidase 15 | **2.1** | 1.8 | 1.2 |
| NM_213826 | USP18 | Ubiquitin specific peptidase 18 | **79.3** | **70.8** | 1.0 |
| NM_001142835 | UTP3 | UTP3, small subunit (SSU) processome component, homolog (S. cerevisiae) | **2.1** | **2.1** | 1.6 |
| NM_001123216 | ZBP1 | Z-DNA binding protein 1 | **8.8** | **10.1** | 0.9 |

**Table S2.** List of down-regulated genes greater than two-fold in NPTr cells infected with *S. suis* strain 31533 (B), swine influenza virus H1N1 (V), or co-infected with *S. suis* and H1N1 (V & B). Values represent mean fold increase in infected-NPTr cells compared to mock-infected NPTr cells (*n* = 4 per group).

| **Genebank ID** | **Gene** | **Gene description** | **V & B** | **V** | **B** |
| --- | --- | --- | --- | --- | --- |
| **Cytokines, chemokines, and related receptors** | | | | | |
| NM_001244546 | CMTM5 | CKLF-like MARVEL transmembrane domain containing 5 | **2.2** | **2.3** | 1.0 |
| NM_214198 | TGFB3 | Transforming growth factor, beta 3 | **4.2** | **3.7** | **2.1** |
| NM_214272 | TGFBR3 | Transforming growth factor, beta receptor III | 1.9 | **2.1** | 1.0 |
| NM_001031775 | TLR1 | Toll-like receptor 1 | **3.0** | 1.6 | 1.3 |
| NM_001030534 | TLR10 | Toll-like receptor 10 | **3.4** | **2.1** | 1.5 |
| NM_213760 | TLR6 | Toll-like receptor 6 | **2.2** | 1.5 | 1.2 |
| **Cytokine signaling** | | | | | |
| XM_001927265 | AKAP12 | A kinase (PRKA) anchor protein 12 | **4.2** | **3.6** | **2.0** |
| NM_001206449 | ANKRD28 | Ankyrin repeat domain 28 | 1.2 | **2.1** | 1.0 |
| XM_003123825 | CAMK4 | Calcium/calmodulin-dependent protein kinase IV | **2.7** | **2.4** | 1.6 |
| XM_003133906 | DAB2 | Disabled homolog 2, mitogen-responsive phosphoprotein (Drosophila) | 1.7 | **2.7** | 1.1 |
| NM_001243663 | EDARADD | EDAR-associated death domain | **2.3** | 1.7 | 1.3 |
| XM_001927175 | EPB49 | Erythrocyte membrane protein band 4.9 (dematin) | **2.4** | **2.3** | 1.4 |
| NM_214292 | EPOR | Erythropoietin receptor | 1.4 | **2.2** | 1.1 |
| XM_001928678 | FGFR1 | Fibroblast growth factor receptor 1 | **2.0** | 1.6 | 1.3 |
| NM_001099924 | FGFR2 | Fibroblast growth factor receptor 2 | **3.0** | **2.5** | 1.1 |
| XM_003123682 | FGFR4 | Fibroblast growth factor receptor 4 | **2.1** | 1.9 | 1.3 |
| NM_001097518 | GNA14 | Guanine nucleotide binding protein (G protein), alpha 14 | **2.7** | **2.4** | 1.3 |
| NM_001134965 | GRB10 | Growth factor receptor-bound protein 10 | **2.7** | **2.8** | 0.8 |
| XM_003131502 | GRB7 | Growth factor receptor-bound protein 7 | **2.3** | **2.5** | 1.6 |
| XM_003123500 | JAK3 | Janus kinase 3 | **3.3** | **3.6** | 1.5 |
| NM_001184946 | LY75 | Lymphocyte antigen 75 | **2.1** | **2.0** | 1.2 |
| NM_001104956 | LY96 | Lymphocyte antigen 96 | **3.1** | **2.5** | 1.6 |
| XM_003133973 | MAP3K1 | Mitogen-activated protein kinase kinase kinase 1 | **2.7** | **2.4** | 1.3 |
| XM_003131293 | MAP3K3 | Mitogen-activated protein kinase kinase kinase 3 | **2.1** | 1.8 | 1.4 |
| NM_001105293 | MUC13 | Mucin 13, cell surface associated | 2.0 | 1.5 | **2.2** |
| NM_001113440 | MUC20 | Mucin 20, cell surface associated | **2.3** | 1.2 | 1.2 |
| XM_001926534 | PIK3IP1 | Phosphoinositide-3-kinase interacting protein 1 | **7.5** | **4.4** | **3.1** |
| NM_213851 | PIK3R5 | Phosphoinositide-3-kinase, regulatory subunit 5 | **2.4** | **2.9** | 1.3 |
| NM_214204 | PKIA | Protein kinase (cAMP-dependent, catalytic) inhibitor alpha | **2.0** | **2.0** | 1.4 |
| NM_001077230 | RIT1 | Ras-like without CAAX 1 | **2.2** | 0.8 | 1.0 |
| NM_001097461 | SOCS2 | Suppressor of cytokine signaling 2 | **2.1** | 1.1 | 1.0 |
| **Defense response** | | | | | |
| NM_214107 | ADM | Adrenomedullin | 1.9 | **2.3** | 1.3 |
| NM_214009 | C3 | Complement component 3 | **2.4** | **2.1** | 1.0 |
| NM_001001646 | C5 | Complement component 5 | **3.0** | **2.6** | 1.7 |
| NM_214301 | CAT | Catalase | **2.1** | **2.0** | 1.3 |
| NM_001101824 | CFB | Complement factor B | **2.6** | **2.5** | 1.3 |
| NM_213838 | DEFB1 | Defensin, beta 1 | **2.7** | **2.1** | 1.1 |
| NM_213999 | DUOX2 | Dual oxidase 2 | **9.6** | **2.3** | **2.3** |
| XM_001929326 | EFNA4 | Ephrin-A4 | **3.7** | **2.6** | 1.3 |
| XM_003132576 | FETUIN | FETUIN protein | **4.8** | **4.7** | 1.6 |
| NM_001115155 | GPX3 | Glutathione peroxidase 3 (plasma) | **2.8** | **2.7** | 1.7 |
| NM_214142 | HCST | Hematopoietic cell signal transducer | 1.7 | **2.0** | 1.1 |
| XM_003481424 | IGSF8 | Immunoglobulin superfamily, member 8 | 1.5 | **2.1** | 1.0 |
| NM_213908 | ITGB2 | Integrin, beta 2 (complement component 3 receptor 3 and 4 subunit) | **2.2** | **2.7** | 1.5 |
| NM_001097424 | ITGB8 | Integrin, beta 8 | **2.3** | **2.3** | 1.3 |
| NM_001037998 | MADCAM1 | Mmucosal vascular addressin cell adhesion molecule 1 | **2.1** | **2.3** | 1.1 |
| XM_003124624 | MGRN1 | Mmahogunin, ring finger 1 | 1.9 | **2.1** | 1.2 |
| NM_001123142 | NCF2 | Neutrophil cytosolic factor 2 | **2.0** | **2.1** | 1.1 |
| XM_003480582 | ORM1 | Orosomucoid 1 | **2.0** | 1.5 | 1.1 |
| NM_001244284 | PGLYRP4 | Peptidoglycan recognition protein 4 | **3.1** | **3.2** | 1.2 |
| XM_003356559 | PRSS16 | Protease, serine, 16 (thymus) | **4.5** | **3.7** | 1.4 |
| XM_001928155 | SIT1 | Signaling threshold regulating transmembrane adaptor 1 | **2.6** | 1.1 | 0.9 |
| NM_001004039 | SLA-DMA | SLA-DM alpha chain | **2.1** | 2.0 | 1.2 |
| NM_001113707 | SLA-DMB | MHC class II, DM beta | **2.3** | 1.8 | 1.3 |
| NM_213870 | SLPI | Secretory leukocyte peptidase inhibitor | **2.6** | 1.9 | 1.5 |
| NM_001044558 | TMEM59 | Transmembrane protein 59 | **2.3** | **2.0** | 1.5 |
| XM_003355308 | UPK3A | Uroplakin 3A | **2.2** | 1.8 | 1.3 |
| **Transcriptional and translational regulation** | | | | | |
| NM_001243420 | CSDC2 | Cold shock domain containing C2, RNA binding | **2.1** | **2.0** | 1.4 |
| NM_001097437 | DNMT3A | DNA (cytosine-5-)-methyltransferase 3 alpha | 1.8 | **2.2** | 1.2 |
| XM_001928532 | HEY1 | Hairy/enhancer-of-split related with YRPW motif 1 | **2.0** | 1.7 | 1.4 |
| NM_001032388 | HNF1A | HNF1 homeobox A | **2.2** | **2.0** | 0.9 |
| NM_001032388 | HNF1B | HNF1 homeobox B | **2.4** | **2.0** | 1.2 |
| NM_001032388 | HNF4A | Hepatocyte nuclear factor 4, alpha | **2.5** | **2.1** | 1.4 |
| NM_001243602 | ID3 | Inhibitor of DNA binding 3, dominant negative helix-loop-helix protein | **2.7** | 1.5 | 1.8 |
| NM_001134346 | KLF11 | Kruppel-like factor 11 | **2.7** | **2.8** | 1.6 |
| NM_001011504 | KLF9 | Kruppel-like factor 9 | **2.1** | **2.2** | **2.1** |
| NM_001038001 | MITF | Microphthalmia-associated transcription factor | **2.1** | **2.0** | 1.2 |
| XM_001927754 | MORF4L1 | Mortality factor 4 like 1 | **3.7** | **3.2** | 1.0 |
| XM_003133181 | MXI1 | MAX interactor 1 | **3.6** | **3.6** | 0.8 |
| NM_001114275 | NCOA2 | Nuclear receptor coactivator 2 | **2.0** | 1.8 | 1.4 |
| XM_003121219 | NCOA7 | Nuclear receptor coactivator 7 | **2.2** | **2.1** | 1.5 |
| NM_214161 | NFATC1 | Nuclear factor of activated T-cells, cytoplasmic, calcineurin-dependent 1 | 1.3 | **2.6** | 0.8 |
| XM_003483682 | NFE2L2 | Nuclear factor (erythroid-derived 2)-like 2 | **2.4** | 1.6 | 1.5 |
| XM_003481738 | NR1H4 | Nuclear receptor subfamily 1, group H, member 4 | 1.2 | **2.2** | 1.1 |
| NM_001190253 | NR2F2 | Nuclear receptor subfamily 2, group F, member 2 | **2.5** | **2.5** | 1.3 |
| XM_001927049 | PDCD4 | Programmed cell death 4 (neoplastic transformation inhibitor) | **2.8** | **2.6** | 1.5 |
| XM_001925111 | PHTF1 | Putative homeodomain transcription factor 1 | **2.1** | **2.3** | 1.4 |
| XM_001926241 | POU3F2 | POU class 3 homeobox 2 | 1.6 | **2.0** | 1.2 |
| NM_213963 | PPARGC-1 | Peroxisome proliferator activated receptor gamma, coactivator 1 alpha | **5.7** | **3.4** | 1.8 |
| XM_003125766 | RFX5 | Regulatory factor X, 5 (influences HLA class II expression) | 1.4 | 1.5 | **2.1** |
| XM_001927928 | RUNX1T1 | Runt-related transcription factor 1; translocated to, 1 (cyclin D-related) | **4.3** | **3.1** | **2.0** |
| NM_213814 | SAL1 | Salivary lipocalin | **2.1** | **2.1** | 1.5 |
| XM_003122960 | SOX-6 | SRY (sex determining region Y)-box 6 | **2.3** | 2.0 | **2.1** |
|  | ZNF395 | Zinc finger protein 395 | **2.1** | **2.2** | 0.7 |
| XM_003131558 | ZNF652 | Zinc finger protein 652 | **2.5** | **2.4** | 1.2 |
| XM_001926173 | ZSWIM3 | Zinc finger, SWIM-type containing 3 | **2.2** | **31.9** | 1.4 |
| **Apoptosis, cell cycle regulation, and oncogenesis** | | | | | |
| NM_001110175 | BCAS1 | Breast carcinoma amplified sequence 1 | **2.7** | **3.8** | 1.5 |
| XM_001927592 | BNIP3L | BCL2/adenovirus E1B 19kDa interacting protein 3-like | **2.0** | **2.0** | 1.1 |
| NM_001097505 | BTG2 | BTG family, member 2 | **2.1** | **2.1** | 1.8 |
| NM_001243354 | DHCR24 | 24-dehydrocholesterol reductase | **2.5** | **2.0** | 1.0 |
| NM_001039749 | DKK3 | Dickkopf 3 homolog (Xenopus laevis) | 1.9 | **2.4** | 1.0 |
| XM_001928760 | EFHC1 | EF-hand domain (C-terminal) containing 1 | 1.8 | **2.0** | 1.1 |
| XM_003357692 | KDM5B | Lysine (K)-specific demethylase 5B | 1.8 | **2.0** | 1.1 |
| NM_001185064 | LYPD1 | LY6/PLAUR domain containing 1 | 1.0 | **2.2** | **2.1** |
| NM_001109944 | PEG10 | Paternally expressed 10 | 1.4 | **2.4** | 1.0 |
| XM_001925030 | PHLPP1 | PH domain and leucine rich repeat protein phosphatase 1 | **2.6** | **2.4** | 1.6 |
| NM_001123109 | S1PR5 | Sphingosine-1-phosphate receptor 5 | **2.3** | **2.1** | 1.3 |
| XM_003358604 | WWTR1 | WW domain containing transcription regulator 1 | **2.2** | **2.9** | 1.4 |
| **Cell adhesion and migration** | | | | | |
| NM_001164006 | AMBP | Alpha-1-microglobulin/bikunin precursor | **6.7** | **7.0** | 1.7 |
| NM_001160083 | CLDN17 | Claudin 17 | **2.2** | **2.4** | 1.2 |
| NM_001160075 | CLDN3 | Claudin 3 | **9.5** | **8.2** | 1.6 |
| NM_001161646 | CLDN8 | Claudin 8 | **3.7** | **4.0** | 1.3 |
| XM_001924325 | COL14A1 | Collagen, type XIV, alpha 1 | **2.9** | **2.2** | 1.3 |
| XM_001926443 | COL8A1 | Collagen, type VIII, alpha 1 | **4.2** | **4.7** | 1.7 |
| XM_001926010 | IGSF1 | Immunoglobulin superfamily, member 1 | **2.8** | **2.4** | 1.2 |
| NM_001246669 | ITGB5 | Integrin, beta 5 | 1.9 | **2.3** | 1.1 |
| NM_214207 | MMP7 | Matrix metallopeptidase 7 (matrilysin, uterine) | **3.9** | **4.0** | 1.2 |
| NM_213907 | PECAM1 | Platelet/endothelial cell adhesion molecule | **4.3** | **3.4** | **2.8** |
| NM_001097494 | PRR2 | Poliovirus receptor related 2 | 1.5 | **2.0** | 1.0 |
| XM_003127747 | PTPRU | Protein tyrosine phosphatase, receptor type, U | 1.6 | **3.0** | 0.9 |
| XM_001926939 | SDC2 | Syndecan 2 | 1.4 | **2.8** | 1.2 |
| NM_001123204 | TN-X | Tenascin-X | **2.7** | **2.6** | 1.0 |
| **Surface receptor molecule, antigen presentation and co-stimulation** | | | | | |
| NM_001008686 | CD3G | CD3g molecule, gamma (CD3-TCR complex) | 1.9 | **2.3** | 1.4 |
| NM_214170 | CD59 | CD59 molecule, complement regulatory protein | **3.5** | **2.8** | 1.6 |
| NM_001145218 | CD82 | CD82 molecule | **2.1** | **2.0** | 1.0 |
| NM_214257 | DPP4 | Dipeptidyl-peptidase 4 | 1.9 | **2.3** | 1.0 |
| NM_214105 | GUCY2C | Guanylate cyclase 2C (heat stable enterotoxin receptor) | **5.0** | **6.3** | 1.5 |
| NM_001244473 | IGF2R | Insulin-like growth factor 2 receptor | **2.1** | 1.2 | 1.0 |
| NM_001142842 | LGALS3 | Lectin, galactoside-binding, soluble, 3 | **2.0** | **2.0** | 1.1 |
| XM_003127883 | MEP1B | Meprin A, beta | **7.8** | **6.5** | **3.2** |
| NM_214176 | OPRL1 | Opiate receptor-like 1 | **2.5** | **2.3** | 0.9 |
| NM_214027 | OXTR | Oxytocin receptor | **2.6** | **2.2** | 1.1 |
| XM_001926786 | P2RY1 | Purinergic receptor P2Y, G-protein coupled, 1 | **2.7** | 1.7 | 1.6 |
| XR_135310 | PIGR | Polymeric immunoglobulin receptor | **8.0** | **5.4** | 2.0 |
|  | THRB | Thyroid hormone receptor beta 1 | 1.0 | **2.0** | 0.9 |
| **Angiogenesis** | | | | | |
| NM_001044573 | ANG1 | Angiogenin | 1.6 | **2.1** | 1.1 |
| NM_001109946 | ANGPTL2 | Angiopoietin-like 2 | **4.1** | **3.9** | 1.5 |
| NM_214120 | F5 | Coagulation factor V (proaccelerin, labile factor) | **2.1** | **2.3** | 1.7 |
| XM_001925764 | PGF | Placental growth factor | 1.9 | **2.4** | 0.7 |
| NM_001078662 | SERPINF1 | Serpin peptidase inhibitor, clade F (alpha-2 antiplasmin, pigment epithelium derived factor), member 1 | **2.0** | **2.1** | 1.2 |
| NM_001130732 | THBD | Thrombomodulin | **2.6** | 1.3 | 1.2 |
| XM_001925442 | VAV3 | Vav 3 guanine nucleotide exchange factor | **2.7** | **2.1** | 1.7 |
| **Cytoskeleton/actin rearrangement** | | | | | |
| XM_001924738 | ADD3 | Adducin 3 (gamma) | **2.2** | **2.1** | 1.4 |
| XM_003121841 | DAAM1 | Dishevelled associated activator of morphogenesis 1 | **2.0** | 1.4 | 1.2 |
| NM_213801 | FGF9 | Fibroblast growth factor 9 (glia-activating factor) | 1.8 | **2.1** | 0.9 |
| XM_003482873 | FRY | Furry homolog (Drosophila) | **3.0** | **2.4** | **2.2** |
| XM_003480596 | GSN | Gelsolin | **2.0** | **2.1** | 1.2 |
| XM_001924292 | KANK1 | KN motif and ankyrin repeat domains 1 | **2.0** | 1.8 | 1.5 |
| NM_001244186 | PSTPIP1 | Proline-serine-threonine phosphatase interacting protein 1 | 1.2 | **2.0** | 1.0 |
| NM_001244417 | RHOV | Ras homolog gene family, member V | **2.5** | **2.9** | 1.0 |
| NM_001114271 | SIRT2 | Sirtuin 2 | **2.1** | 1.8 | 1.1 |
| XM_003480228 | SYNE1 | Spectrin repeat containing, nuclear envelope 1 | **2.8** | **3.3** | 1.7 |
| **Lipid metabolism** | | | | | |
| XM_003361841 | ACAD10 | Acyl-CoA dehydrogenase family, member 10 | **2.1** | 1.5 | 1.2 |
| NM_213897 | ACADL | Acyl-CoA dehydrogenase, long chain | **2.0** | **2.0** | 1.3 |
| XM_001929040 | ACOT4 | Acyl-CoA thioesterase 4 | **2.0** | **2.1** | 1.2 |
| XM_003132281 | ACOX2 | Acyl-CoA oxidase 2, branched chain | **2.6** | **2.6** | 1.4 |
| NM_001002801 | APOC3 | Apolipoprotein C-III | **2.3** | **2.6** | 1.3 |
| NM_001040640 | APOM | Apolipoprotein M | **2.3** | **2.8** | 1.6 |
| XM_001928792 | AUH | AU RNA binding protein/enoyl-CoA hydratase | 1.4 | **2.0** | 1.2 |
| NM_214246 | CES1 | Carboxylesterase 1 | 1.3 | **2.1** | 1.0 |
| NM_001243217 | CROT | Carnitine O-octanoyltransferase | 1.3 | **2.1** | 1.0 |
| XM_003125510 | ENPP2 | Ectonucleotide pyrophosphatase/phosphodiesterase 2 | 1.8 | **2.1** | 1.3 |
| NM_001004046 | FABP1 | Fatty acid binding protein 1, liver | **2.4** | **2.2** | 1.1 |
| NM_001025229 | FABP7 | Fatty acid binding protein 7, brain | 1.8 | **2.3** | 1.0 |
| NM_001004049 | HSD3B1 | Hydroxy-delta-5-steroid dehydrogenase, 3 beta- and steroid delta-isomerase 1 | **2.9** | **2.3** | 1.3 |
| NM_001123134 | LIPA | Lipase A, lysosomal acid, cholesterol esterase | **2.1** | **2.0** | 1.3 |
| NM_001130734 | LPIN1 | Lipin 1 | 0.9 | **2.0** | 0.7 |
| XM_003131589 | PDK2 | Pyruvate dehydrogenase kinase, isozyme 2 | **2.1** | **2.2** | 1.3 |
| NM_001159306 | PDK4 | Pyruvate dehydrogenase kinase, isozyme 4 | **2.4** | **2.6** | **2.1** |
| XM_003483312 | PLA1A | Phospholipase A1 member A | **2.2** | 1.4 | **2.1** |
| XM_003481683 | PLBD1 | Phospholipase B domain containing 1 | **2.5** | **2.9** | 0.9 |
| NM_001160082 | PNPLA4 | Patatin-like phospholipase domain containing 4 | **2.4** | **2.3** | 1.3 |
| NM_001123167 | PPT2 | Palmitoyl-protein thioesterase 2 | 1.5 | **2.1** | 1.2 |
| NM_001114278 | SCD5 | Stearoyl-CoA desaturase 5 | **5.5** | **5.8** | 1.9 |
| NM_001199890 | VLDLR | Very low density lipoprotein receptor | **2.5** | **3.0** | 1.7 |
| **Biological and metabolic processes** | | | | | |
| NM_001244900 | AASS | Aminoadipate-semialdehyde synthase | **3.4** | **4.3** | 1.6 |
| XM_003124739 | ABCA3 | ATP-binding cassette, sub-family A (ABC1), member 3 | **2.5** | **2.9** | 1.3 |
| XM_001927577 | ABHD4 | Abhydrolase domain containing 4 | 1.9 | **2.1** | 1.1 |
| NM_001123070 | ACE2 | Angiotensin I converting enzyme (peptidyl-dipeptidase A) 2 | **2.7** | **3.1** | **2.2** |
| NM_214209 | ACP5 | Acid phosphatase 5, tartrate resistant | **3.0** | **3.5** | **2.0** |
| NM_001243547 | AGPHD1 | Aminoglycoside phosphotransferase domain containing 1 | **2.1** | 2.0 | 1.1 |
| NM_001011693 | AGRP | Agouti related protein homolog (mouse) | 2.0 | **2.4** | 1.2 |
| NM_001244884 | AGXT2 | Alanine--glyoxylate aminotransferase 2 | **2.6** | **2.6** | **2.3** |
| XM_003482314 | AK7 | Adenylate kinase 7 | **3.2** | **3.4** | 1.4 |
| NM_001044618 | AKR1C1 | Aldo-keto reductase family 1, member C1 (dihydrodiol dehydrogenase 1; 20-alpha (3-alpha)-hydroxysteroid dehydrogenase) | **2.9** | **2.9** | **2.5** |
| NM_001123075 | AKR1C4 | Aldo-keto reductase family 1, member C4 (chlordecone reductase; 3-alpha hydroxysteroid dehydrogenase, type I; dihydrodiol dehydrogenase 4) | **2.2** | **2.5** | 1.2 |
| NM_001038626 | AKR1CL1 | Aldo-keto reductase family 1, member C-like 1 | **2.1** | **2.1** | 1.1 |
| NM_001243928 | ALDOC | Aldolase C, fructose-bisphosphate | 1.7 | **2.0** | 0.6 |
| NM_214195 | AMY2 | Amylase, alpha 2B (pancreatic) | 1.7 | **2.0** | **2.0** |
| XM_003481037 | ANKS4B | Ankyrin repeat and sterile alpha motif domain containing 4B | **6.7** | **3.2** | 1.9 |
| NM_214277 | ANPEP | Alanyl (membrane) aminopeptidase | **2.3** | **2.4** | 0.7 |
| XM_003130763 | ARMC4 | Armadillo repeat containing 4 | **2.2** | **2.4** | 1.2 |
| NM_213933 | ARSA | Arylsulfatase A | **2.1** | **2.0** | 1.3 |
| XM_001929642 | ARVCF | Armadillo repeat gene deleted in velocardiofacial syndrome | **2.3** | **2.1** | 1.2 |
| XM_001925519 | ATP8B4 | ATPase, class I, type 8B, member 4 | **3.5** | **3.4** | **2.2** |
| NM_001114672 | ATP9A | ATPase, class II, type 9A | **3.2** | **3.1** | 1.4 |
| XM_003483558 | ATRNL1 | Attractin-like 1 | **2.6** | **2.8** | 1.3 |
| NM_213952 | AVP | Arginine vasopressin | **2.4** | **2.3** | 1.4 |
| XM_003122909 | BBOX1 | Butyrobetaine (gamma), 2-oxoglutarate dioxygenase (gamma-butyrobetaine hydroxylase) 1 | **3.3** | **3.2** | 1.5 |
| XM_003353833 | BEST1 | Bestrophin 1 | 2.0 | **2.0** | 1.3 |
| NM_001004038 | CA11 | Carbonic anhydrase XI | **2.2** | **2.2** | 1.1 |
| XM_001924554 | CAR | Constitutive androstane receptor | **2.8** | **2.6** | 1.9 |
| NM_214073 | CBR1 | Carbonyl reductase 1 | **2.5** | **2.3** | 1.5 |
| XM_003128860 | CC2D2A | Coiled-coil and C2 domain containing 2A | **3.9** | **2.7** | 1.6 |
| XM_001924211 | CCDC37 | Coiled-coil domain containing 37 | **2.2** | **2.0** | 1.4 |
| NM_001243301 | CCPG1 | Cell cycle progression 1 | **2.2** | **2.3** | 1.4 |
| XM_003362154 | CDH16 | Cadherin 16, KSP-cadherin | **5.7** | **6.1** | 1.7 |
| NM_214139 | CLC-5 | Outwardly rectifying chloride channel | **2.1** | 1.9 | 1.3 |
| XM_003359358 | CNNM2 | Cyclin M2 | **2.7** | **2.6** | 1.8 |
| XM_003132473 | CP | Ceruloplasmin (ferroxidase) | **3.2** | **3.9** | 1.9 |
| NM_213881 | CRY | CRY protein | 1.7 | **2.1** | 1.2 |
| XM_003481589 | CSAD | Cysteine sulfinic acid decarboxylase | **2.3** | **2.2** | 1.3 |
| XM_003122495 | CTSF | Cathepsin F | **2.1** | **2.2** | 1.2 |
| NM_214302 | CTSK | Cathepsin K | **2.1** | **2.6** | 1.4 |
| XM_001929214 | CUL7 | Cullin 7 | **2.8** | **2.8** | 1.4 |
| NM_001128452 | CYBRD1 | Cytochrome b reductase 1 | **2.4** | **2.4** | 1.2 |
| NM_214427 | CYP11A1 | Cytochrome P450, family 11, subfamily A, polypeptide 1 | **2.3** | **2.3** | **2.2** |
| NM_214414 | CYP2C33 | Cytochrome P450 2C33 | **2.2** | **2.2** | **2.4** |
| XM_003359304 | CYP2C34 | Cytochrome P450 2C34 | **2.2** | **2.2** | **2.1** |
| NM_214420 | CYP2C49 | Cytochrome P450 2C49 | 1.9 | 1.7 | **2.1** |
| NM_001101027 | CYP39A1 | Cytochrome P450, family 39, subfamily A, polypeptide 1 | **2.4** | **2.7** | 1.8 |
| NM_214423 | CYP3A29 | Cytochrome P450 3A29 | **2.3** | 1.6 | 1.8 |
| NM_214422 | CYP3A39 | Cytochrome P450 3A39 | **3.2** | **2.8** | **2.5** |
| NM_001134824 | CYP3A46 | Cytochrome P450 3A46 | **2.1** | 1.8 | **2.2** |
| NM_001005352 | CYP7A1 | Cytochrome P450, family 7, subfamily A, polypeptide 1 | **4.2** | **2.3** | **2.1** |
| NM_214066 | DAO1 | D-amino acid oxidase | **4.4** | **3.8** | **2.8** |
| NM_001025227 | DCT | Dopachrome tautomerase (dopachrome delta-isomerase, tyrosine-related protein 2) | **2.3** | **2.2** | **2.0** |
| NM_213854 | DDC | Dopa decarboxylase (aromatic L-amino acid decarboxylase) | **5.0** | **4.6** | **2.1** |
| XM_001926085 | DEPTOR | DEP domain containing MTOR-interacting protein | **4.7** | **3.9** | 1.6 |
| XM_003133509 | DFNB59 | Deafness, autosomal recessive 59 | **2.4** | **2.1** | 1.1 |
| NM_001001627 | DIO1 | Deiodinase, iodothyronine, type I | **6.6** | **6.5** | **2.4** |
| NM_001244667 | DNAL4 | Dynein, axonemal, light chain 4 | **2.2** | **2.1** | 1.5 |
| NM_001123107 | DOK5 | Docking protein 5 | **3.4** | **3.0** | 1.7 |
| XM_003360844 | DPF3 | D4, zinc and double PHD fingers, family 3 | **2.4** | **3.0** | **2.5** |
| NM_001243550 | DUOXA2 | Dual oxidase maturation factor 2 | **2.5** | **2.7** | 1.6 |
| NM_001044607 | ECH1 | Enoyl CoA hydratase 1, peroxisomal | **2.0** | **2.0** | 1.4 |
| XM_003358734 | EHHADH | Enoyl-CoA, hydratase/3-hydroxyacyl CoA dehydrogenase | **4.1** | **3.3** | 1.3 |
| NM_001044527 | ENO3 | Enolase 3 (beta, muscle) | **2.0** | **2.0** | 1.2 |
| XM_001926345 | ENO4 | Enolase family member 4 | **3.2** | **3.5** | 1.6 |
| NM_214153 | ENTPD1 | Ectonucleoside triphosphate diphosphohydrolase 1 | **2.0** | **2.5** | 1.5 |
| NM_214355 | EPHX1 | Epoxide hydrolase 1, microsomal (xenobiotic) | **4.5** | **3.8** | 1.8 |
| XM_001924217 | ERMP1 | Endoplasmic reticulum metallopeptidase 1 | **2.9** | **2.5** | 1.5 |
| XM_003129835 | EXPH5 | Exophilin 5 | **3.5** | **3.6** | 1.8 |
| XM_003356212 | EXTL1 | Exostoses (multiple)-like 1 | 1.5 | **2.3** | 0.9 |
| NM_001243549 | EXTL2 | Exostoses (multiple)-like 2 | **2.2** | **2.0** | 1.3 |
| NM_001243630 | FAM166B | Family with sequence similarity 166, member B | **5.3** | **5.3** | **2.1** |
| NM_213979 | FBP1 | Fructose-1,6-bisphosphatase 1 | **4.8** | **4.9** | **2.1** |
| NM_001244497 | FBXO21 | F-box protein 21 | **2.3** | **2.1** | 1.6 |
| NM_001005152 | FGL2 | Fibrinogen-like 2 | **2.6** | **5.0** | 0.9 |
| XM_001928594 | FMO5 | Flavin containing monooxygenase 5 | **2.2** | **2.4** | 1.5 |
| NM_214384 | FOLH1 | Folate hydrolase (prostate-specific membrane antigen) 1 | **3.3** | **3.7** | 1.5 |
| NM_213830 | FOLR1 | Folate receptor 1 (adult) | **4.1** | **4.1** | 0.6 |
| NM_213853 | FOLR2 | Folate receptor 2 (fetal) | **3.8** | **3.9** | 1.6 |
| NM_001204852 | FPGT | Fucose-1-phosphate guanylyltransferase | **2.1** | 1.8 | 1.6 |
| NM_214275 | FTCD | Formiminotransferase cyclodeaminase | **3.2** | **3.0** | 1.6 |
| XM_001928787 | FZD3 | Frizzled family receptor 3 | **2.4** | **2.2** | 1.2 |
| NM_001244429 | GAL3ST1 | Galactose-3-O-sulfotransferase 1 | **2.5** | **2.4** | 1.1 |
| NM_001206447 | GALNTL2 | UDP-N-acetyl-alpha-D-galactosamine:polypeptide N-acetylgalactosaminyltransferase-like 2 | **3.3** | **3.4** | 1.4 |
| NM_001128442 | GATM | Glycine amidinotransferase (L-arginine:glycine amidinotransferase) | **2.2** | **2.4** | **2.3** |
| XM_003356971 | GC | Group-specific component (vitamin D binding protein) | **2.7** | **2.1** | 1.2 |
| XM_003354890 | GCKR | Glucokinase (hexokinase 4) regulator | **2.5** | **2.3** | 1.5 |
| NM_001244397 | GFAP | Glial fibrillary acidic protein | **2.0** | 1.9 | 1.2 |
| XM_001925457 | GLT8D1 | Glycosyltransferase 8 domain containing 1 | 1.7 | **2.0** | 1.3 |
| NM_001244093 | GNPDA1 | Glucosamine-6-phosphate deaminase 1 | **2.9** | **2.4** | 1.6 |
| NM_001098584 | GPNMB | Glycoprotein (transmembrane) nmb | **2.1** | **2.0** | 1.2 |
| NM_001206355 | GPR110 | G protein-coupled receptor 110 | **4.4** | **10.0** | 0.9 |
| XM_001928511 | GPR116 | G protein-coupled receptor 116 | **2.4** | 1.6 | 1.1 |
| XM_003354582 | GPRC5B | G protein-coupled receptor, family C, group 5, member B | **2.3** | **2.5** | 1.3 |
| XM_003126361 | GRIP1 | Glutamate receptor interacting protein 1 | **2.5** | **2.8** | 1.5 |
| NM_214389 | GSTA1 | Glutathione S-transferase alpha 1 | **3.1** | **3.6** | 1.3 |
| XM_001929370 | GSTT1 | Glutathione S-transferase theta 1 | **2.2** | **2.1** | 1.2 |
| NM_001243690 | HABP2 | Hyaluronan binding protein 2 | **7.1** | **4.0** | **3.1** |
| NM_213921 | HEXB | Hexosaminidase B (beta polypeptide) | 1.7 | **2.1** | 1.1 |
| NM_001243656 | HIST1H2BD | Histone cluster 1, H2bd | **2.7** | **2.9** | 0.8 |
| XM_001928882 | HKDC1 | Hexokinase domain containing 1 | **2.3** | **2.3** | 1.0 |
| NM_001098587 | HPS1 | Hermansky-Pudlak syndrome 1 | **2.0** | 2.0 | 1.4 |
| NM_001098588 | HPS3 | Hermansky-Pudlak syndrome 3 | **2.3** | **2.1** | 1.3 |
| XM_001925492 | HS2ST1 | Heparan sulfate 2-O-sulfotransferase 1 | **2.1** | **2.0** | 1.2 |
| NM_214248 | HSD11B1 | Hydroxysteroid (11-beta) dehydrogenase 1 | 1.8 | **2.0** | 0.9 |
| XM_001929463 | HVCN1 | Hydrogen voltage-gated channel 1 | **2.1** | **2.0** | 1.3 |
| NM_214441 | HYAL1 | Hyaluronoglucosaminidase 1 | **3.0** | 1.5 | 1.2 |
| NM_213847 | ICA | Porcine inhibitor of carbonic anhydrase | **2.9** | **2.8** | 1.4 |
| XM_003132384 | IFT122 | Intraflagellar transport 122 homolog (Chlamydomonas) | **2.0** | 1.6 | 1.1 |
| XM_003132513 | IFT80 | Intraflagellar transport 80 homolog (Chlamydomonas) | 1.7 | **2.0** | 1.5 |
| XM_001927370 | IGFBP1 | Insulin-like growth factor binding protein 1 | **3.5** | **4.6** | **2.2** |
| NM_001244470 | IHH | Indian hedgehog | **4.1** | **4.0** | 1.1 |
| XM_003127958 | INADL | InaD-like (Drosophila) | **2.1** | **2.0** | 1.4 |
| XM_003132192 | IP6K2 | Inositol hexakisphosphate kinase 2 | **2.0** | 1.7 | 1.9 |
| XM_003358505 | ITPR1 | Inositol 1,4,5-trisphosphate receptor, type 1 | **3.1** | **3.8** | 1.5 |
| NM_214416 | IYD | Iodotyrosine deiodinase | **2.2** | **2.2** | 1.3 |
| NM_001244551 | KAZALD1 | Kazal-type serine peptidase inhibitor domain 1 | **2.8** | **2.3** | 1.6 |
| XM_003134741 | KCND2 | Potassium voltage-gated channel, Shal-related subfamily, member 2 | **2.2** | 1.8 | 1.6 |
| NM_214093 | KCNE3 | Potassium voltage-gated channel, Isk-related family, member 3 | 1.0 | **3.4** | 1.0 |
| XM_003122329 | LCN5 | Lipocalin 5 | **2.3** | **2.2** | 1.2 |
| XM_003359266 | LDB3 | LIM domain binding 3 | **2.1** | **2.1** | 0.9 |
| XM_003122906 | LGR4 | Leucine-rich repeat containing G protein-coupled receptor 4 | **2.4** | **2.8** | 1.3 |
| NM_214080 | LHB | Luteinizing hormone beta polypeptide | **3.2** | **2.7** | 1.7 |
| NM_001105291 | LNPEP | Leucyl/cystinyl aminopeptidase | **2.1** | 1.6 | 1.4 |
| XM_003135348 | LONRF3 | LON peptidase N-terminal domain and ring finger 3 | **3.4** | **2.2** | 1.5 |
| NM_001206403 | LOX | Lysyl oxidase | **2.3** | **2.7** | 1.2 |
| NM_001244357 | LPAR1 | Lysophosphatidic acid receptor 1 | **2.2** | 1.7 | 1.2 |
| XM_001929519 | LRRC71 | Leucine rich repeat containing 71 | **2.9** | **2.3** | 1.1 |
| XM_003356432 | LRRIQ3 | Leucine-rich repeats and IQ motif containing 3 | **2.3** | **2.3** | 1.7 |
| NM_001243339 | MAT1A | Methionine adenosyltransferase I, alpha | **2.2** | 0.8 | 0.7 |
| XM_003355304 | MCOLN3 | Mucolipin 3 | **2.5** | **2.2** | 1.2 |
| NM_214300 | MGST1 | Microsomal glutathione S-transferase 1 | **2.0** | **2.1** | 1.1 |
| NM_001243709 | MORN3 | MORN repeat containing 3 | **4.3** | **3.8** | 1.6 |
| XM_003127575 | MTHFR | Methylenetetrahydrofolate reductase (NAD(P)H) | **2.4** | **2.2** | 1.0 |
| XM_003131577 | MYCBPAP | MYCBP associated protein | **2.0** | 1.9 | 1.4 |
| NM_213855 | MYH7 | Myosin, heavy chain 7, cardiac muscle, beta | **2.4** | **2.2** | **2.2** |
|  | N4BP2L1 | NEDD4 binding protein 2-like 1 | **2.1** | **2.4** | 1.3 |
| NM_001097520 | NAGS | N-acetylglutamate synthase | **2.7** | **2.1** | 1.0 |
|  | ND5 | NADH dehydrogenase subunit 5 | 1.5 | **2.0** | 1.0 |
| XM_003483124 | NEK11 | NIMA (never in mitosis gene a)- related kinase 11 | **2.1** | **2.3** | 1.3 |
| NM_001143720 | NME4 | Non-metastatic cells 4, protein expressed in | **2.3** | **2.3** | 1.1 |
| XM_003124830 | NPHP1 | Nephronophthisis 1 (juvenile) | **2.0** | 1.9 | 1.3 |
| XM_001929587 | NPR1 | Natriuretic peptide receptor A/guanylate cyclase A (atrionatriuretic peptide receptor A) | **2.6** | **2.2** | 0.9 |
| NM_001244322 | NPR2 | Natriuretic peptide receptor B/guanylate cyclase B (atrionatriuretic peptide receptor B) | 1.6 | **2.0** | 1.2 |
| NM_001122996 | OAZ3 | Ornithine decarboxylase antizyme 3 | **2.2** | **2.1** | 1.4 |
| NM_214094 | OCA2 | Oculocutaneous albinism II | **4.9** | **5.3** | 1.6 |
| NM_213938 | OXCT1 | 3-oxoacid CoA transferase 1 | **2.3** | **2.3** | 1.3 |
| XM_003123844 | P311 | P311 protein | **2.0** | **2.3** | 1.2 |
| XM_001926022 | PANK1 | Pantothenate kinase 1 | **5.1** | **4.6** | 0.9 |
| XM_003482286 | PAPLN | Papilin, proteoglycan-like sulfated glycoprotein | **2.8** | **3.0** | 1.5 |
| NM_214349 | PC | Pyruvate carboxylase | **2.8** | **2.5** | 1.2 |
| NM_001244484 | PCDH7 | Protocadherin 7 | 1.5 | **2.5** | 0.8 |
| NM_001123158 | PCK1 | Phosphoenolpyruvate carboxykinase 1 (soluble) | **6.8** | **4.7** | 1.7 |
| XM_001928875 | PCMTD1 | Protein-L-isoaspartate (D-aspartate) O-methyltransferase domain containing 1 | **2.3** | **2.6** | 1.6 |
| XM_001928893 | PCOLCE2 | Procollagen C-endopeptidase enhancer 2 | 1.4 | **2.0** | 0.9 |
| XM_003129933 | PDZD3 | PDZ domain containing 3 | **3.4** | **4.1** | 1.6 |
| XM_001928171 | PDZK1 | PDZ domain containing 1 | **14.0** | **15.0** | **3.0** |
| NM_001001769 | PDZK1IP1 | PDZK1 interacting protein 1 | **2.1** | 1.6 | 1.3 |
| XM_003358425 | PFKFB4 | 6-phosphofructo-2-kinase/fructose-2,6-biphosphatase 4 | **2.8** | **2.3** | 1.2 |
| NM_001244594 | PHEX | Phosphate regulating endopeptidase homolog, X-linked | 1.0 | **2.0** | 1.0 |
| NM_001123162 | PHGDH | Phosphoglycerate dehydrogenase | **3.5** | **3.4** | 1.5 |
| NM_001244581 | PHYHIPL | Phytanoyl-CoA 2-hydroxylase interacting protein-like | **2.0** | **2.2** | 1.1 |
|  | PLEKHH2 | Pleckstrin homology domain containing, family H (with MyTH4 domain) member 2 | **2.1** | **2.1** | 1.8 |
| XM_001928589 | PM20D2 | Peptidase M20 domain containing 2 | **2.2** | **2.5** | 1.3 |
| NM_001184895 | PMM1 | Phosphomannomutase 1 | **2.0** | **4.0** | 1.4 |
| NM_001184895 | PPIL6 | Peptidylprolyl isomerase (cyclophilin)-like 6 | **4.5** | **3.8** | 1.7 |
| NM_214025 | PPP2R2B | Protein phosphatase 2, regulatory subunit B, beta | **2.5** | **2.0** | 1.7 |
| NM_001100192 | PPP2R5A | Protein phosphatase 2, regulatory subunit B', alpha | **2.1** | **2.1** | 1.3 |
| NM_214252 | PRM2 | Protamine 2 | **3.0** | **3.3** | 1.5 |
| NM_001123174 | RAB22A | RAB22A, member RAS oncogene family | **3.3** | **3.6** | 0.9 |
| NM_001123179 | RAB3A | RAB3A, member RAS oncogene family | 1.6 | **2.0** | 1.1 |
| NM_214199 | RAMP1 | Receptor (G protein-coupled) activity modifying protein 1 | **2.1** | **2.3** | **23.8** |
| XM_001926447 | RASGEF1A | RasGEF domain family, member 1A | **2.9** | **3.0** | 1.5 |
| NM_214451 | RBP2 | Retinol binding protein 2, cellular | **2.6** | **2.4** | 1.4 |
| XM_001927535 | RCCD1 | RCC1 domain containing 1 | 1.9 | **2.0** | 1.3 |
| XM_001928082 | RDH10 | Retinol dehydrogenase 10 (all-trans) | **2.5** | **2.4** | 1.6 |
| NM_001243402 | RDH12 | Retinol dehydrogenase 12 (all-trans/9-cis/11-cis) | **3.0** | **2.6** | 1.7 |
| NM_001243866 | RDH16 | Retinol dehydrogenase 16 (all-trans) | **2.5** | **2.5** | 1.6 |
| NM_001077220 | RGN | Regucalcin (senescence marker protein-30) | **3.6** | **3.2** | **2.1** |
| XM_001925440 | RGS22 | Regulator of G-protein signaling 22 | **2.7** | **2.4** | 1.3 |
| NM_213996 | RHBG | Rh family, B glycoprotein (gene/pseudogene) | **2.6** | **2.5** | 1.0 |
| NM_001172369 | ROPN1 | Rhophilin associated tail protein 1 | **3.3** | **3.4** | 1.5 |
| XM_001926970 | RSPH3 | Radial spoke 3 homolog (Chlamydomonas) | **2.0** | 1.6 | 1.4 |
| XM_003480267 | RSPH4A | Radial spoke head 4 homolog A (Chlamydomonas) | **4.3** | **4.8** | **3.0** |
| NM_001129963 | RTN4 | Reticulon 4 | **2.2** | **2.1** | 0.8 |
| XM_003131351 | RUNDC3A | RUN domain containing 3A | **2.1** | **2.1** | 1.2 |
| NM_001123193 | SADC | Arginine decarboxylase | 1.4 | **2.1** | 1.5 |
| NM_214079 | SAG | S-antigen; retina and pineal gland (arrestin) | **3.4** | **4.3** | 1.9 |
| XM_001929498 | SARDH | Sarcosine dehydrogenase | **4.8** | **3.7** | 1.0 |
| NM_001105287 | SCG5 | Secretogranin V (7B2 protein) | **2.2** | **2.9** | 1.3 |
| NM_001134823 | SEPP1 | Selenoprotein P, plasma, 1 | **3.2** | **3.2** | 1.8 |
| XM_003483544 | SFXN3 | Sideroflexin 3 | **2.0** | 1.8 | 1.1 |
| XM_001929079 | SH3GL3 | SH3-domain GRB2-like 3 | 1.5 | **2.3** | 1.1 |
| NM_001244340 | SH3YL1 | SH3 domain containing, Ysc84-like 1 (S. cerevisiae) | **2.4** | **3.0** | 1.3 |
|  | SHC3 | SHC (Src homology 2 domain containing) transforming protein 3 | 1.7 | **2.2** | 0.6 |
| NM_001097514 | SLC15A2 | Solute carrier family 15 (H+/peptide transporter), member 2 | **2.1** | **2.1** | 1.2 |
| NM_001128445 | SLC16A1 | Solute carrier family 16, member 1 (monocarboxylic acid transporter 1) | **2.0** | 1.6 | 1.3 |
| XM_001928811 | SLC16A12 | Solute carrier family 16, member 12 (monocarboxylic acid transporter 12) | **5.0** | **3.9** | 1.7 |
| NM_213902 | SLC22A2 | Solute carrier family 22 (organic cation transporter), member 2 | **3.0** | **2.9** | 1.1 |
| NM_001135966 | SLC26A11 | Solute carrier family 26, member 11 | **2.8** | **2.5** | 1.5 |
| NM_001130248 | SLC26A3 | Solute carrier family 26, member 3 | **2.8** | **3.4** | **3.3** |
| NM_001083931 | SLC27A1 | Solute carrier family 27 (fatty acid transporter), member 1 | **2.1** | **2.0** | 0.8 |
| XM_003480364 | SLC27A2 | Solute carrier family 27 (fatty acid transporter), member 2 | **7.9** | **13.7** | 1.8 |
| XM_003132985 | SLC2A11 | Solute carrier family 2 (facilitated glucose transporter), member 11 | **3.2** | **2.6** | 1.1 |
| NM_001044623 | SLC34A1 | Solute carrier family 34 (sodium phosphate), member 1 | **3.8** | **2.0** | 1.3 |
| XM_003353725 | SLC34A3 | Solute carrier family 34 (sodium phosphate), member 3 | **2.6** | **2.3** | 1.2 |
| NM_001123042 | SLC3A1 | Solute carrier family 3 (cystine, dibasic and neutral amino acid transporters, activator of cystine, dibasic and neutral amino acid transport), member 1 | **2.1** | **2.3** | 1.3 |
| NM_001101818 | SLC44A4 | Solute carrier family 44, member 4 | **2.5** | **2.6** | 1.3 |
| NM_001243471 | SLC46A3 | Solute carrier family 46, member 3 | **3.6** | **2.8** | 1.6 |
| NM_001164021 | SLC5A1 | Solute carrier family 5 (sodium/glucose cotransporter), member 1 | **2.4** | **3.1** | **2.0** |
| NM_001012297 | SLC5A10 | Solute carrier family 5 (sodium/glucose cotransporter), member 10 | **3.0** | **3.3** | 1.2 |
| NM_001110421 | SLC7A7 | Solute carrier family 7 (amino acid transporter light chain, y+L system), member 7 | **7.1** | **11.1** | **2.2** |
| NM_001110171 | SLC7A9 | Solute carrier family 7 (glycoprotein-associated amino acid transporter light chain, bo,+ system), member 9 | **3.2** | **2.1** | 1.8 |
| XM_003480256 | SMPDL3A | Sphingomyelin phosphodiesterase, acid-like 3A | **2.1** | **2.2** | 1.0 |
| NM_001078687 | SMPX | Small muscle protein, X-linked | **2.3** | 1.4 | 0.9 |
| XM_001929226 | SNX33 | Sorting nexin 33 | **2.2** | 2.0 | 1.2 |
| NM_001044561 | SPEF2 | Sperm flagellar 2 | **2.6** | **2.0** | 1.2 |
| NM_001037150 | SULT2A1 | Sulfotransferase family, cytosolic, 2A, dehydroepiandrosterone (DHEA)-preferring, member 1 | **2.8** | 1.8 | 1.2 |
| XM_003353166 | SYTL3 | Synaptotagmin-like 3 | **4.3** | **5.0** | 1.0 |
| NM_001113288 | TAS1R3 | Taste receptor, type 1, member 3 | **5.3** | **4.9** | **2.1** |
| XM_003131144 | TBC1D16 | TBC1 domain family, member 16 | **2.0** | **2.2** | 1.1 |
| NM_001032356 | TCTEX1D4 | Tctex1 domain containing 4 | **4.3** | **3.8** | 1.5 |
| XM_003361599 | TET1 | Tet methylcytosine dioxygenase 1 | **3.2** | **2.9** | 1.9 |
| NM_001244653 | TF | Transferrin | **3.6** | **3.1** | **2.1** |
| XM_001924775 | TMEM66 | Transmembrane protein 66 | **2.0** | 1.5 | 1.2 |
| NM_001243407 | TMX4 | Thioredoxin-related transmembrane protein 4 | **2.0** | 1.8 | 1.4 |
| NM_213912 | TNNI1 | Troponin I type 1 (skeletal, slow) | **2.8** | **2.4** | 1.0 |
| NM_001098599 | TNNI3 | Troponin I type 3 (cardiac) | **2.8** | **2.7** | 1.6 |
| XM_003354608 | TNP2 | Transition protein 2 | **2.8** | 0.9 | 1.0 |
| NM_001244691 | TRIM55 | Tripartite motif containing 55 | **2.3** | 1.3 | 0.9 |
| XM_003121515 | TRPM7 | Transient receptor potential cation channel, subfamily M, member 7 | **2.3** | **2.0** | 1.5 |
| XM_003134594 | TRPV6 | Transient receptor potential cation channel, subfamily V, member 6 | **8.6** | **6.9** | 1.4 |
| XM_003126923 | TSNAXIP1 | Translin-associated factor X interacting protein 1 | **2.6** | 1.9 | 1.4 |
| NM_001243860 | TSPAN1 | Tetraspanin 1 | **3.7** | **3.7** | 1.4 |
| XM_001926303 | TSTD1 | Thiosulfate sulfurtransferase (rhodanese)-like domain containing 1 | **2.7** | **2.1** | 1.5 |
| NM_001025226 | TYRP1 | Tyrosinase-related protein 1 | **2.8** | **3.0** | 1.6 |
| XM_003121566 | UBR1 | Ubiquitin protein ligase E3 component n-recognin 1 | **2.2** | **2.2** | 1.6 |
| XM_001928551 | ULK1 | Unc-51-like kinase 1 (C. elegans) | **2.4** | **2.0** | 1.4 |
| NM_001244219 | UROC1 | Urocanase domain containing 1 | **2.5** | **2.5** | 1.3 |
| XM_003122977 | USP47 | Ubiquitin specific peptidase 47 | **2.6** | **2.1** | 1.2 |
|  | VWA7 | Von Willebrand factor A domain containing 7 | **2.6** | **3.5** | 1.4 |
| NM_214383 | ZAN | Zonadhesin | **2.2** | **2.8** | 1.1 |
| XM_003124806 | ZC3H6 | Zinc finger CCCH-type containing 6 | 1.7 | **2.0** | 1.6 |

**Table S3.** Validation of microarray gene expression using real-time RT-qPCR in NPTr cells infected with *S. suis* strain 31533 (B), swine influenza virus H1N1 (V), or co-infected with *S. suis* and H1N1 (V & B)

| **Gene** | **RNA quantification method** | **V & B** | **V** | **B** |
| --- | --- | --- | --- | --- |
| *Vcam* | Microarray | 3.9 | 2.7 | 0.8 |
|  | qRT-PCR | 5.0 | 3.5 | 0.7 |
| *Plau* | Microarray | 20.2 | 8.6 | 2.1 |
|  | qRT-PCR | 22.6 | 9.8 | 2.2 |
| *Ifnβ1* | Microarray | 73.0 | 62.2 | 0.9 |
|  | qRT-PCR | 382.2 | 347.8 | 0.8 |
| *Il1α* | Microarray | 6.9 | 4.3 | 2.1 |
|  | qRT-PCR | 5.8 | 3.0 | 1.8 |
| *Tnf* | Microarray | 1.5 | 1.3 | 1.1 |
|  | qRT-PCR | 11.3 | 9.8 | 0.8 |
| *Il6* | Microarray | 7.9 | 7.5 | 1.1 |
|  | qRT-PCR | 15.9 | 12.6 | 1.1 |
| *Il8* | Microarray | 23.4 | 12.3 | 1.3 |
|  | qRT-PCR | 18.8 | 10.0 | 0.8 |
| *Cox2* | Microarray | 23.6 | 3.5 | 2.4 |
|  | qRT-PCR | 121.0 | 15.4 | 5.3 |
| *Irf1* | Microarray | 0.9 | 3.1 | 2.5 |
|  | qRT-PCR | 0.6 | 2.4 | 2.1 |
| *Irf7* | Microarray | 1.3 | 12.5 | 9.5 |
|  | qRT-PCR | 0.7 | 12.0 | 7.9 |
| *Ccl5* | Microarray | 144.2 | 122.7 | 6.8 |
|  | qRT-PCR | 102.4 | 68.9 | 4.9 |
| *Il12a* | Microarray | 1.4 | 2.2 | 2.6 |
|  | qRT-PCR | 0.7 | 1.1 | 1.1 |
| *Ifnλ1* | Microarray | 5.5 | 5.4 | 0.8 |
|  | qRT-PCR | 3.4 | 5.0 | 0.4 |
